# Chloramphenicol stress triggers oxidative adaptation in *Acinetobacter baumannii* ATCC19606 devoid of RND efflux pumps AdeAB or AdeIJ

**DOI:** 10.1128/msystems.00009-26

**Published:** 2026-09-03

**Authors:** Wuen Ee Foong, Xinxin Xiang, Wenjun He, Xuan Yan, Jiabin Huang, Klaas M. Pos, Heng-Keat Tam

**Affiliations:** 1 Department of Medical Microbiology, Hunan Provincial Key Laboratory for Special Pathogens Prevention and Control, Hengyang Medical School, University of South China 159373, Hengyang, Hunan, China; 2 Department of Biochemistry and Molecular Biology, Hengyang Medical School, University of South China, Hengyang, Hunan, China; 3 National Health Commission Key Laboratory of Birth Defect Research and Prevention, Hunan Provincial Maternal and Child Health Care Hospital, Changsha, Hunan, China; 4 Institute of Medical Microbiology, Virology and Hygiene, University Medical Center Hamburg-Eppendorf, Martinistraße, Hamburg, Germany; 5 Institute of Biochemistry, Goethe-University Frankfurt 399040, Frankfurt am Main, Germany; Universita degli Studi di Bari Aldo Moro, Bari, Italy; University of Health and Allied Sciences, Ho, Volta Region, Ghana

**Keywords:** *Acinetobacter baumannii*, stress adaptation, CraA, RND, antibiotic resistance

## Abstract

Efflux pumps play a key role in both intrinsic and acquired antibiotic resistance in *Acinetobacter baumannii*, yet their broader physiological roles remain unclear. Here, we investigated the transcriptomic and phenotypic responses of *A. baumannii* ATCC19606 mutants lacking the efflux pumps AdeAB, AdeIJ, or CraA under chloramphenicol stress. The deletion of *craA* resulted in a 32-fold reduction in chloramphenicol MIC, while Δ*adeIJ* showed a modest 4-fold decrease, and Δ*adeAB* had no effect on chloramphenicol susceptibility. Transcriptomic profiling revealed minimal alterations in Δ*craA*, but notable transcriptional reprogramming in Δ*adeAB* and Δ*adeIJ*, including upregulation of ribosomal genes, iron-sulfur cluster biogenesis, aromatic compound catabolism, and amino acid transport systems. Under chloramphenicol stress, Δ*adeAB* exhibited metabolic remodeling, activating oxidative stress defenses and protein quality control pathways while repressing type VI secretion. Both Δ*adeIJ* and wild-type strains upregulated arginine and glutamate metabolism, likely contributing to redox balance. Phenotypically, the Δ*adeIJ* strain showed elevated basal and H₂O₂-induced ROS levels, as well as heightened sensitivity to nitrosative stress and Cu^2+^, which may suggest a role for AdeIJ in oxidative and metal stress responses. Despite increased *adeAB* and *craA* expression under chloramphenicol stress, only *craA* deletion significantly impaired resistance, highlighting its dominant role in chloramphenicol efflux. Collectively, our findings reveal efflux pump-specific and strain-specific adaptations to antibiotic stress, positioning CraA as a major determinant of chloramphenicol resistance, while RND transporters such as AdeABC and AdeIJK may also be associated with broader stress adaptation processes, including redox and metal homeostasis, in *A. baumannii*.

**IMPORTANCE:**

Efflux pumps are key drivers of multidrug resistance in *Acinetobacter baumannii*, yet their broader roles in stress adaptation remain insufficiently understood. Here, we show that the loss of major efflux systems reshapes the transcriptomic and metabolic landscape under chloramphenicol stress, a condition that also imposes oxidative stress. In particular, the RND efflux pump AdeIJK may be linked to alterations in cellular responses to oxidative, nitrosative, and metal stress, although the mechanistic basis of this interplay remains to be further investigated. Overall, these findings suggest that efflux pumps may contribute to bacterial resilience beyond drug resistance, providing additional insight into efflux system hierarchy and functional redundancy in *A. baumannii*, and may inform future studies aimed at understanding persistence and efflux-mediated multidrug resistance mechanisms.

## INTRODUCTION

*Acinetobacter baumannii* is a resilient gram-negative aerobic coccobacillus that manifests multidrug-resistant (MDR) phenotypes, particularly thriving in clinical environments. Infections associated with *A. baumannii* primarily affect debilitated patients, leading to ventilator-associated pneumonia, bloodstream infections, urinary tract infections, meningitis, and wound infections, conditions often associated with high morbidity and mortality rates (1). The persistence of *A. baumannii* is largely attributed to its MDR capabilities and its remarkable adaptability to diverse environmental conditions. Genomic plasticity plays a critical role in this adaptability, enabling the acquisition of various resistance genes and contributing to the widespread emergence of MDR *A. baumannii* strains (2). Key contributors to *A. baumannii* resistance include its restrictive outer membrane and diverse families of MDR efflux pumps (3, 4). The global spread of MDR and even pandrug-resistant *A. baumannii* poses a formidable challenge to contemporary medicine.

Chloramphenicol, a bacteriostatic antibiotic, inhibits bacterial protein synthesis by reversibly binding to the peptidyltransferase center of the 50S ribosomal subunit, thereby blocking tRNA attachment at the ribosomal A-site (5). To exert its antimicrobial effect on gram-negative bacteria, chloramphenicol must traverse both outer and inner membranes to reach the cytoplasm, entering via outer membrane porins and crossing the inner membrane by passive diffusion or active transport via YdgR transporter (6–8). Due to potential side effects, chloramphenicol is typically reserved for severe, life-threatening infections (9). Nevertheless, extensive use in clinical and agricultural settings has led to rising resistance levels. In clinical MDR isolates, resistance to chloramphenicol is commonly mediated by enzymatic inactivation via chloramphenicol acetyltransferases or through active efflux mechanisms (10, 11). In *A. baumannii*, however, resistance is predominantly attributed to active efflux mediated by the resistance-nodulation-division-type (RND) efflux pumps AdeABC and AdeIJK, as well as the major facilitator superfamily (MFS) efflux pump CraA (12–14), together with reduced outer membrane permeability, rather than enzymatic inactivation (15). Expression of *adeABC* and *adeIJK* is controlled by transcriptional regulators such as AdeRS and AdeN (16, 17), whereas the regulatory mechanisms governing *craA* remain less clearly defined, although its expression appears condition-dependent and inducible by both nutrient composition and chloramphenicol exposure (14, 18).

Recent studies highlight that antibiotic efficacy is closely tied to bacterial metabolic state, with antibiotic-induced metabolic dysregulation playing a significant role in determining susceptibility (19). Thus, bacterial resistance is not solely governed by genetic determinants, but also by metabolic activity, stress responses, and phenotypic plasticity (20–22). These non-genetic, transient resistance mechanisms, often termed "adaptive resistance,” allow bacteria to survive transient antibiotic exposure and may precede the development of stable, long-term resistance (23, 24). Despite extensive research into classical drug–target interactions, the intrinsic response of *A. baumannii* to antibiotics remains poorly understood. In light of the growing threat of antimicrobial resistance and the looming prospect of a post-antibiotic era, a deeper understanding of intrinsic tolerance mechanisms is critical for developing new strategies to combat *A. baumannii* infections.

To examine adaptive antibiotic response to chloramphenicol, we performed RNA-seq on wild-type *A. baumannii* ATCC19606 and mutants lacking the AdeAB or AdeIJ. Chloramphenicol was selected as a model antibiotic because prior studies identified AdeABC, AdeIJK, and CraA as major resistance determinants in this strain (12–14). While *adeIJK* is conserved across the *Acinetobacter* genus, suggesting a broader physiological function, *adeABC* is variably distributed (25, 26), pointing to distinct evolutionary trajectories. Supporting this functional divergence, previous work in strains AB5075 and AYE showed that *adeB* inactivation alters motility, competence, type IV pilus genes, iron acquisition, and biofilm-associated pathways, including poly-β-1,6-N-acetyl-D-glucosamine (*pga*) biosynthesis (27). Similarly, disruption of *adeIJK* has been linked to changes in motility, *pga*-dependent biofilm formation, lipid synthesis, efflux networks, and fatty acid, iron, and carbon metabolism genes (27). Given their overlapping substrate profiles but divergent evolutionary histories, we hypothesized that deletion of these efflux pumps would trigger strain-specific resistance adaptations under chloramphenicol stress. Consistent with this broader physiological role of RND pumps, our data show that *craA* is expressed at low basal levels in wild-type ATCC19606 strain, whereas Δ*adeAB* and Δ*adeIJ* mutants display distinct regulatory and metabolic remodeling under chloramphenicol stress. Together, these findings suggest that efflux system perturbation may coincide with changes in metabolic and stress-response networks during adaptation to antibiotic stress in *A. baumannii*.

## MATERIALS AND METHODS

### Bacterial strains and growth conditions

All *A. baumannii* strains (Table S1) were stored at −80°C in 20% glycerol and cultured by inoculation into sterile LB. *E. coli* S17-1 λpir donor strains harboring the plasmid pMo130-Tel^R^_cloned-fragments were cultured in LB supplemented with 50 mg/L kanamycin.

### Construction of *astA* and transporter genes knockout

Briefly, targeted gene deletion mutants (e.g., *abaQ*, *adeAB*, *adeIJ*, *astA*, *craA*, and *mumT*) were generated by allelic exchange using suicide plasmids carrying upstream and downstream homologous regions, followed by antibiotic selection and sucrose counter-selection as previously described (28, 29). PCR products of upstream and downstream homologous region of the *adeAB*, *adeIJ*, or *craA* genes were cloned into pBIISK_sacB/kanR by Gibson assembly. The resulting plasmids were electroporated into *A. baumannii* cells using a MicroPulser System (program Ec1; Bio-Rad Laboratories, Hercules, CA), and subsequently, transformants were selected on LB agar + 50 mg/L kanamycin. Upstream and downstream homologous regions of the *abaQ*, *astA*, and *mumT* genes were cloned into pMo130-Tel^R^ by Gibson assembly. Conjugation was performed by incubating the mixture of *E. coli* S17-1 donor strain harboring the plasmid pMo130-Tel^R^_target-gene and the *A. baumannii* strain on LB agar at 30°C for 24 h. The conjugants were resuspended in sterile LB and plated onto LB agar supplemented with 30 mg/L tellurite and 50 mg/L ampicillin. Counter-selection was performed on LB supplemented with 15% sucrose. Correct gene replacement was first verified by PCR and Sanger sequencing across the recombination junctions. All primers used are listed in Table S1.

### Whole-genome sequencing

Whole-genome sequencing was performed to confirm the genetic integrity of *A. baumannii* ATCC19606 wild-type and the isogenic deletion mutants Δ*adeAB*, Δ*adeIJ*, and Δ*craA*. Genomic DNA from each strain was fragmented to an average size of ~350 bp using a Covaris LE220R-plus system (Covaris, USA), followed by end repair, A-tailing, adapter ligation, and PCR amplification. Libraries were sequenced on Illumina platforms (150 bp paired-end) by Novogene Bioinformatics Technology Co., Ltd. (Beijing, China).

Reads were quality-trimmed using Trimmomatic v0.39 (30) and assembled *de novo* with SPAdes v3.15.5 (31). The assemblies comprised 22, 40, 38, and 35 contigs (>500 bp) for WT, Δ*adeAB*, Δ*adeIJ*, and Δ*craA*, respectively. Contigs with significant similarity to plasmid sequences, identified by BLASTn searches against the NCBI nucleotide database, were excluded from chromosomal reconstruction (32, 33). The remaining contigs were scaffolded using RagTag (34).

For variant analysis, reads from each mutant were mapped to the wild-type assembly and were analyzed independently using Snippy v4.6.0 (https://github.com/tseemann/snippy) and freebayes v1.3.6 (35), retaining only SNPs and InDels identified by both pipelines. Structural variants were detected through pairwise assembly comparison using MUMmer (36) and Assemblytics (37).

### Drug agar plate assay

Drug susceptibility assays were performed as previously described (28). Briefly, overnight-grown cell cultures of *A. baumannii* ATCC19606 wild-type (control) and knockout strains (including RND mutants, Δ*craA*, Δ*abaQ*, Δ*astA*, and Δ*mumT*) were diluted to OD_600_ 10^0^–10^−5^, and 3 μL of the diluted cultures were spotted on LB agar plate supplemented with chloramphenicol. Drug-free LB medium served as the control. Plates were incubated at 37°C overnight.

### MIC measurement

Minimum inhibitory concentration (MIC) of chloramphenicol was conducted using the microdilution method (38). Approximately 25 μL of overnight cultures of *A. baumannii* ATCC19606 wild-type (control) and the efflux pump knockout strains (OD_600_ of 0.5) were inoculated into 4 mL of serial 2-fold chloramphenicol dilutions in LB. Drug-free LB medium served as the control. Cultures were incubated for 18 h at 37°C with shaking at 130 rpm. The readout of OD_600_ at 16 h was used to calculate the 50% inhibitory concentration of growth (IC_50_).

### Growth curve analysis

Growth curve analysis was performed as previously described (39). Briefly, overnight cell cultures of *A. baumannii* wild-type (control) and efflux pump knockout strains were diluted in fresh LB to an OD_600_ of 0.02. For each strain, 30 μL of diluted cultures were inoculated into 120 μL of LB supplemented with chloramphenicol (final concentration = 8 and 16 μg/mL), 0.5 mM H_2_O_2_, 1.25 mM copper sulfate, manganese chloride (final concentration = 4 and 8 mM), or 32 mM sodium nitroprusside (SNP) in a U-bottom 96-well polystyrene plate. Drug-free LB medium served as the control. Cells were grown for 16 h at 37°C with agitation (linear amplitude: 2 mm), and OD_600_ was measured every 20 min using the Tecan Infinite M200 Pro Microplate Reader (Tecan, Männedorf, Switzerland).

IC_50_ was estimated by fitting the data to a four-parameter logistic model using “dr4pl” package in R 4.4.1 (40). Each of the bacterial growth efflux curves was individually analyzed using the “Wrangle and Analyze Growth Curve Data” method (“gcplyr” package) in R 4.4.1 (41). Each of the bacterial growth parameters, including maximum growth rates (μ_max_), lag time, the minimum time required to reach μ_max_ (*T*_μmax_), doubling time (*T*_*d*_), the maximum OD_600_ at 16 h (OD_max_), and area under the growth curves (AUC), were analyzed between wild-type and efflux pump knockout strains using a linear mixed-effects model by maximum likelihood, with a random intercept for each experiment, according to the equation parameter ~0 + genotype + (1 | exp), implemented using “lmerTest” package in R 4.4.1 (42), where parameter represents individual bacterial growth parameters, genotype denotes the *A. baumannii* strains, and exp corresponds to independent biological replicates. Pairwise comparisons of growth parameters of wild-type and knockout strains were analyzed using general linear hypotheses and Tukey’s multiple comparison test with the “multcomp” package in R 4.4.1 (43).

### Hydrogen peroxide disc diffusion assay

H_2_O_2_ disc diffusion assays were performed as previously described (44), with minor modifications. Briefly, overnight cultures of *A. baumannii* wild-type (control) and efflux pump knockout strains were diluted into fresh LB to an OD_600_ of 0.05 and grown to mid-log phase (OD_600_ = 0.6–0.8). For each strain, 1 mL of culture (adjusted to OD_600_ of 0.6 or 0.06 as indicated) was mixed with 15 mL of molten soft LB agar (0.75% agar) and overlaid onto LB agar plates. Sterile 6 mm filter paper discs were placed on the solidified agar surface and loaded with 10 μL of 2.5% H₂O₂. Plates were incubated at 25°C or 37°C for 16–18 h. Since the knockout strains consistently showed reduced susceptibility to H₂O₂ compared to the wild-type strain, we analyzed the data using a linear mixed-effects model with maximum likelihood estimation, incorporating a random intercept for each biological replicate, as previously described (14). The model was defined as “diameter ~0 + genotype + (1 | exp)” and implemented using the the “lme4” package in R 4.4.1 (45), where diameter represents the measured inhibition zone, genotype denotes the *A. baumannii* strains, and exp corresponds to independent biological replicates.

### Reactive oxygen species (ROS) assay

ROS assay was performed as previously described with slight modification (46). Briefly, bacterial cultures, including wild-type (control) and efflux pump knockout strains, were grown in LB at 37°C with shaking to an OD_600_ of 0.6, harvested by centrifugation, and washed twice with Dulbecco’s phosphate-buffered saline (DPBS; 8 g/L NaCl, 0.2 g/L KCl, 1.44 g/L Na_2_HPO_4_.2H_2_O, 0.2 g/L KH_2_PO_4_, 10 mM MgCl_2_). Cell pellets were resuspended in DPBS containing 100 µM 2′,7′-dichlorodihydrofluorescein diacetate (DCFH-DA) to an OD_600_ of 1.0. Following incubation for 30 min at 37°C with shaking at 750 rpm, cells were washed with DPBS, and 150 µL of the suspension was transferred to wells containing either H_2_O_2_ (20 mM final concentration) or DMSO as the solvent control for DCFH-DA. The plate was incubated at 37°C with linear shaking (2 mm amplitude), and fluorescence (excitation 485 nm, emission 535 nm) was measured at 10-minute intervals over 3 h using Tecan Infinite M200 Pro Microplate Reader (Tecan, Switzerland). The fluorescence curves were fitted to a logistic model (equation 1) using non-linear least squares regression (“nls” package) in R 4.4.1:


(1) $$y=\frac{A}{1+\exp\left(-\frac{t-t_{1/2}}{s}\right)}$$

where *y* denotes the fluorescence intensity of 2′,7′-dichlorodihydrofluorescein, *t* is time, *A* is the upper asymptote, *S* defines the curve steepness, and *t*_1/2_ represents the time at which fluorescence reaches half of the maximum asymptote.

### Ethidium efflux assays and statistical analysis

Ethidium efflux assays were performed as previously described (28), with minor modifications. Overnight cultures of *A. baumannii* wild-type (control) and efflux pump knockout strains were inoculated into 20 mL fresh LB broth at an initial OD_600_ of 0.05 and grown to mid-log phase (OD_600_ = 0.6–0.8). Cells were harvested, washed twice with DPBS, and resuspended in the same buffer to OD_600_ = 1.0. To dissipate the electrochemical gradient, cells were incubated with 40 μM of carbonyl cyanide *m*-chlorophenyl hydrazone (CCCP) at 37°C for 10 min, followed by incubation with 10 μM ethidium bromide for 30–45 min to allow dye accumulation. Cells were then washed twice with DPBS and resuspended in fresh buffer. Efflux was initiated by the addition of glucose to a final concentration of 0.36%, and fluorescence was monitored using a Tecan Microplate Reader (Tecan, Switzerland) at excitation and emission wavelengths of 535 and 610 nm, respectively.

Individual ethidium efflux curves were analyzed using non-linear least squares regression with the Levenberg-Marquardt algorithm implemented in the “nls.multstart” package in R 4.4.1 (14, 47). Data were fitted to a sigmoid model (equation 2), with a fixed upper asymptote (*y* = 1), where *H* represents the log-transformed time required to remove half of the accumulated ethidium (relative fluorescence ≈ 0.5), *S* defines the curve steepness, and *Y_0_* corresponds to the lower asymptote.


(2) $$y(t,H,S,Y_{0})=1-\frac{1-Y_{0}}{1+\exp\left(\frac{H-\log t}{S}\right)}$$

Statistical comparisons were performed using a linear mixed-effects model fitted by restricted maximum likelihood (REML) with a random intercept structure. The model was defined as “*H* ~0 + genotype + (1 | exp/rep)” and implemented using the the “lme4” package in R 4.4.1 (45), where *H* denotes the log-transformed half-efflux time derived from equation 2, genotype represents the respective *A. baumannii* strains, *Exp* corresponds to independent biological experiments, and rep denotes technical replicates nested within each experiment. Pairwise comparisons between genotypes were conducted using general linear hypothesis testing with Tukey's multiple comparison correction in the “multcomp” package (43).

### RNA extraction

Overnight cultures of *A. baumannii* ATCC19606 wild-type, ∆*adeAB*, ∆*adeIJ*, and ∆*craA* were inoculated into 50 mL of LB supplemented with subinhibitory concentration (Table 1) of chloramphenicol to an initial OD_600_ of 0.05. Cultures were incubated at 37°C with 130 rpm to an OD_600_ of 0.5–0.7. For the untreated samples (control), cells were grown in LB without chloramphenicol to the same growth phase. Cell suspensions of 500 μL were collected and treated with the RNAprotect Bacteria Reagent (Qiagen, Hilden, Germany). RNA extraction was conducted using RNeasy Mini Kit (Qiagen, Hilden, Germany). DNA was removed by on-column DNA digestion and treatment with Turbo DNase (Thermo Fisher Scientific, Waltham, MA). RNA samples were purified by RNeasy MinElute Cleanup kit (Qiagen, Hilden, Germany). The concentration and the quality of the RNA samples were determined using Agilent 2100 Bioanalyzer (Agilent Technologies, Santa Clara, CA). These RNA samples were subsequently used for reverse transcription quantitative PCR (RT-qPCR) and RNA sequencing.

**TABLE 1:** MIC and IC_50_ of *A. baumannii* ATCC19606 of wild-type (WT) and efflux pump knockout strains (∆*adeAB*, ∆*adeIJ*, and ∆*craA*) in the presence of chloramphenicol or CuSO_4_^*b*^

Strain	MIC CuSO_4_ (mM)	Chloramphenicol		
		IC_50_ (μM)^*a*^	MIC (mg/L)	Sub-MIC (mg/L)^*c*^
WT	2.5	46.23	64	8
∆*craA*	2.5	**2.77**	**2**	0.5
∆*adeAB*	2.5	**40.33**	64	8
∆*adeIJ*	2.5	**23.59**	**16**	4

^*a*^ Tukey’s multiple comparison test between efflux pump knockout strains compared to WT: *P* < 0.001.

^*b*^ Boldface values indicate significant differences in IC₅₀ and MIC relative to the wild-type strain (WT).

^*c*^ Sub-MIC indicates the concentration of chloramphenicol used for the analysis of gene expression.

### Reverse transcription qPCR

One-step RT-qPCR was performed on a thermal cycler Rotor-Gene Q (Qiagen, Hilden, Germany) using QuantiNova SYBR Green RT-PCR kit (Qiagen, Hilden, Germany) using the primers listed as shown previously (48) in Table S1, with *rpoB* as reference gene. Gene expression was analyzed as previously described (38). The Δ*C*_*T*_ values were calculated as *C*_*T*(rpoB)_ – *C*_*T*(efflux pump)_. Mean differences in Δ*C*_*T*_ values between “antibiotic treatment” groups and “control” groups, or between “gene knockout” groups and “control” group, were analyzed using an ANOVA model in R version 4.4.1 with Gene and Gene:Treatment interaction terms.

### Library construction, RNA sequencing, and quality control

Bacterial ribosomal RNA was removed from the total RNA using the Ribo-Zero kit and then precipitated with ethanol. After random mRNA fragmentation, the first-strand cDNA was synthesized using random hexamer primers. RNase H, DNA polymerase I, dNTPs, and dUTPs were added in the second-strand synthesis buffer (Illumina, San Diego, CA) to initiate the second-strand synthesis. The construction of double-stranded cDNA libraries was completed after a series of terminal repair, A-tailing, sequencing adapter ligation, size selection, and PCR enrichment. The quality of libraries was assessed using Qubit 2.0 and real-time PCR for quantification and Agilent 2100 Bioanalyzer for size distribution detection. RNA sequencing was performed using Novogene in-house protocol on the Illumina NovaSeq platform (HWI-ST1276) (Illumina, San Diego, CA) with paired-end 150 bp sequencing strategy (Novogene Co. Ltd., Beijing, China).

In total, 18 RNA-seq samples were processed using the nf-core framework for RNA-seq analysis (nf-core/rnaseq v1.3dev) to obtain raw counts (49), with CP059040 (*Acinetobacter baumannii* strain ATCC 19606) as the reference genome. The nf-core pipeline offers multiple alignment options, with HISAT2 (v2.1.0) selected for this analysis (50). FeatureCounts was then employed to assign reads to genomic features and quantify gene expression levels (51). The raw RNA-seq counts were normalized and variance-stabilized using the regularized log transformation (rlog) from DESeq2 (52), which reduces the variance-over-mean trend. DESeq2 was subsequently used to identify differentially expressed genes (DEGs) between conditions, with genes considered significantly enriched if they exhibited a |log_2_ fold change| ≥1.2 and an adjusted *P*-value <0.05. Normalized rlog counts were used for downstream clustering analysis and visualization. Principal component analysis (PCA) and sample distance heatmaps confirmed high reproducibility across all biological replicates. Gene enrichment analysis was performed using the clusterProfiler package in R (53), incorporating Gene Ontology (GO) and Kyoto Encyclopedia of Genes and Genomes (KEGG) pathway analyses to identify molecular functions, biological processes, cellular components, and metabolic pathways significantly associated with the DEGs. UpSet plots were generated in R 4.4.1 using “ComplexUpset” package (54).

## RESULTS

### Structural variant analysis confirms targeted gene deletions

Structural variant analysis confirmed a single, targeted deletion in each engineered strain relative to wild type. In Δ*adeAB*, a 4,282-bp deletion (positions 1844323–1848605; CP059040) removes nearly the entire *adeAB* operon, encompassing *adeA* (H0N29_08675; 1844319–1845509) and *adeB* (H0N29_08680; 1845506–1848616). The deletion begins 4 bp within *adeA* and terminates 11 bp upstream of the *adeB* stop codon. In Δ*adeIJ*, a 4,443-bp deletion (positions 737224–741667; CP059040) removes the reverse-strand *adeIJ* operon, including *adeJ* (H0N29_03545; complement 737233–740409) and *adeI* (H0N29_03550; complement 740422–741672). The breakpoints are located 9 bp upstream of *adeJ* and 5 bp before the end of *adeI*. In Δ*craA*, a 1,157-bp deletion (positions 364652–365809; CP059040) removes the central region of the *craA* coding sequence (H0N29_01695; 364609–365838), spanning from 43 bp downstream of the start codon to 29 bp upstream of the stop codon. Consensus variant analysis using Snippy and freebayes detected no additional SNPs or small InDels outside the intended deletions, confirming that each mutant differs from wild type only at the targeted locus.

### Chloramphenicol susceptibility in efflux pump knockout strains of *A. baumannii*

**Fig 1:**
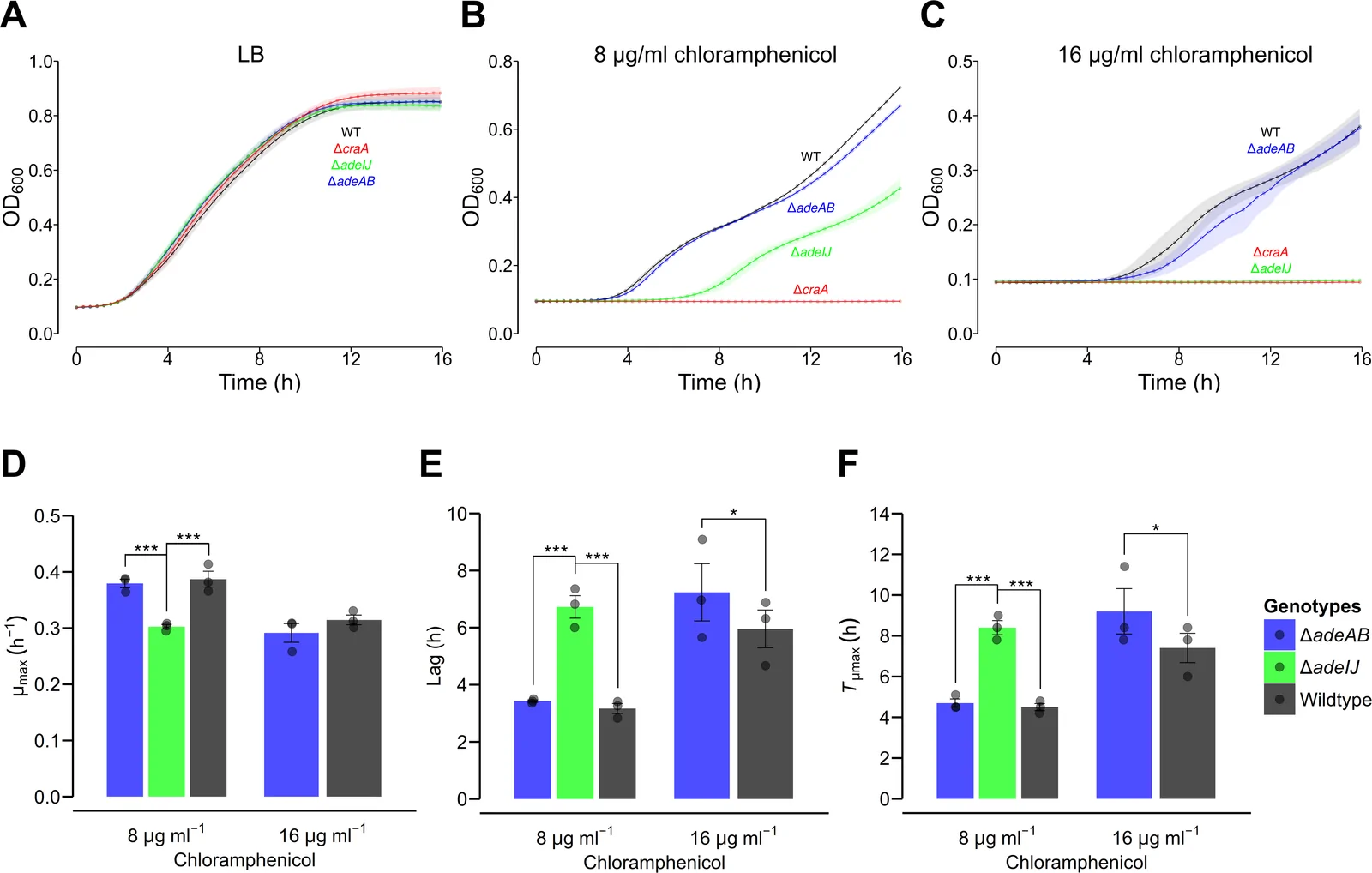
Growth kinetics of *A. baumannii* ATCC19606 of wild-type (WT) and efflux pump knockout strains (∆*adeAB*, ∆*adeIJ*, and ∆*craA*) in the presence of chloramphenicol. (**A–C**) Growth kinetic analysis of *A. baumannii* ATCC19606 wild-type and efflux pump knockout strains in liquid LB medium containing (**A**) 0 µg/mL, (**B**) 8 µg/mL, or (**C**) 16 µg/mL chloramphenicol. Data represent means ± SEM from at least three independent biological replicates. (**D–F**) Growth parameters of *A. baumannii* WT and knockout strains cultured in LB medium supplemented with chloramphenicol: (**D**) maximum growth rates (μ_max_, h^−1^); (**E**) lag time (h); and (**F**) time to reach μ_max_ (*T*_μmax_, h). Data represent individual biological replicates (black dots) and the mean ± SEM from at least three independent biological experiments. Statistical significance was determined using general linear hypothesis testing followed by Tukey’s multiple comparison test. ***P < 0.001; *P < 0.05.

To determine appropriate concentrations of chloramphenicol for downstream gene expression profiling, we measured MIC of chloramphenicol for wild-type ATCC19606 and its efflux pump knockout strains (Δ*adeAB*, Δ*adeIJ*, and Δ*craA*). Growth curve analysis showed no significant growth defects among the strains when cultured in LB medium in absence of antibiotic (Fig. 1A). MIC analysis revealed significant susceptibility differences among the strains in the presence of chloramphenicol, with the Δ*adeIJ* and Δ*craA* strains exhibiting a 4-fold and 32-fold reduction in MIC, respectively, compared to the wild-type strain (Table 1). Growth assays in LB medium supplemented with chloramphenicol demonstrated pronounced susceptibility of Δ*craA* (MIC = 2 µg/mL, IC_50_ = 2.77 µM) and moderate susceptibility of Δ*adeIJ* (MIC = 16 µg/mL, IC_50_ = 23.59 µM) (Fig. 1B; Table 1; Fig. S1A). In contrast, deletion of *adeAB* did not alter susceptibility to chloramphenicol (MIC = 64 µg/mL for both Δ*adeAB* and wild type) (Table 1). Both wild-type and Δ*adeAB* strains exhibited similar maximum growth rates (μ_max_) when treated with 16 μg/mL chloramphenicol (wild type: 0.31 h⁻¹; Δ*adeAB*: 0.29 h⁻¹) (Fig. 1C and D). However, subtle yet reproducible differences were observed in other growth metrics, such as IC₅₀ (wild type: 46.23 μM; Δ*adeAB*: 40.33 μM), lag time (wild type: 5.95 h; Δ*adeAB*: 7.24 h), and time to reach μ_max_ (wild type: 7.4 h; Δ*adeAB*: 9.2 h) (Fig. 1C, E, and F; Table 1; Fig. S1A). Consistent with previous observations (14), the Δ*craA* mutant exhibited a modest but statistically significant reduction in ethidium efflux activity compared with the wild-type and RND knockout strains (Fig. S1B and C), supporting its functional contribution to multidrug efflux. The Δ*adeIJ* mutant likewise displayed a smaller yet significant reduction in ethidium efflux relative to the wild type (Fig. S1C). Together, these findings suggest that although *adeAB* inactivation does not affect MIC, it induces mild growth attenuation in response to chloramphenicol, indicating a degree of antibiotic-induced physiological stress.

### Gene expression of efflux pumps

**Fig 2:**
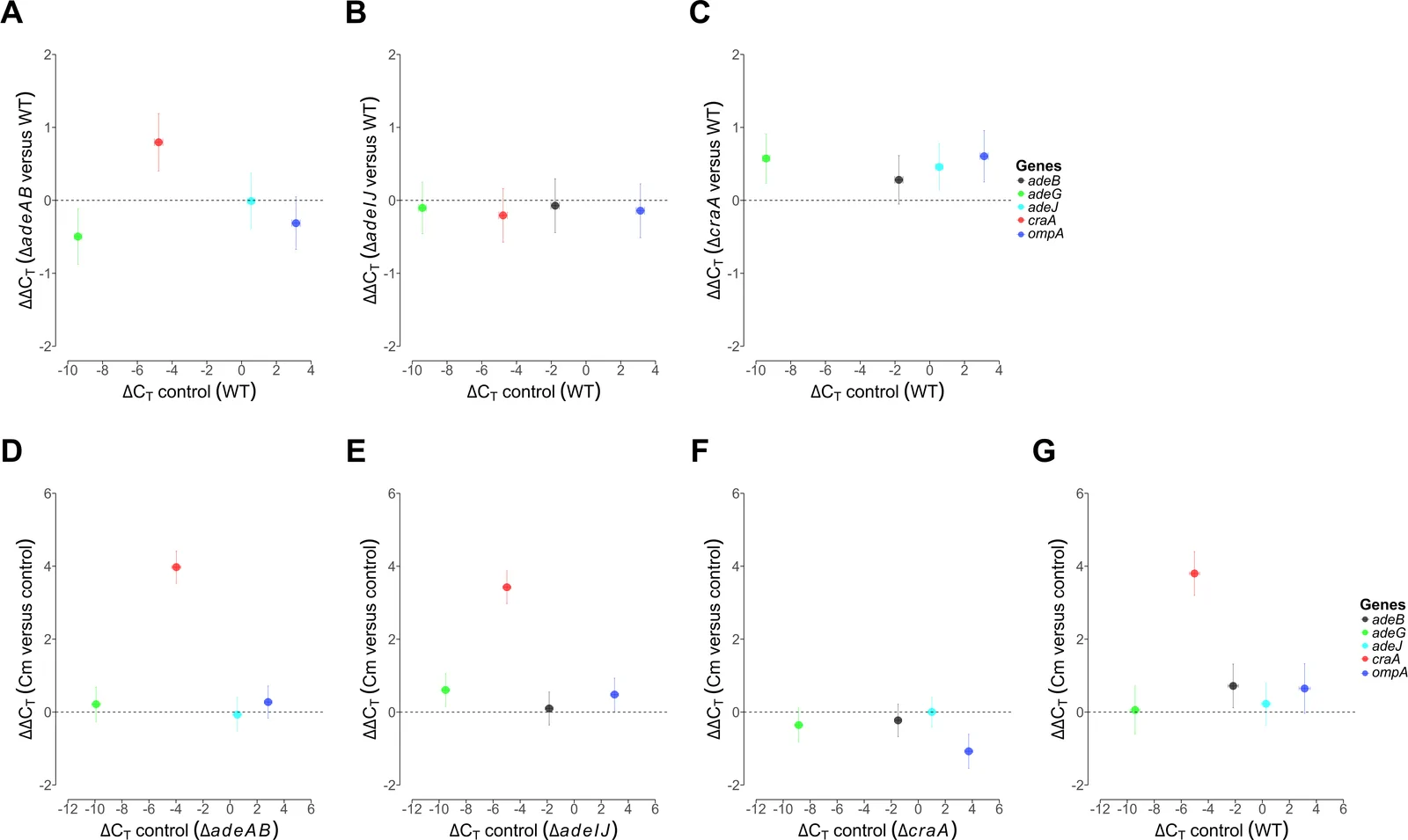
Gene expression analysis of *craA*, *ompA*, and RND efflux pump genes in *A. baumannii* ATCC19606. (**A–C**) Expression profiles in knockout strains relative to the wild type (WT): (**A**) Δ*adeAB*, (**B**) Δ*adeIJ*, and (**C**) Δ*craA*. For these panels, the Δ*C*_*T*_ values on the *x*-axis correspond to the Δ*C*_*T*_ of the wild-type control; therefore, identical baseline values are shown for comparison across strains. (**D–G**) Gene expression of efflux pump genes in the absence or presence of chloramphenicol (Cm): (**D**) Δ*adeAB*, (**E**) Δ*adeIJ*, (**F**) Δ*craA*, and (**G**) WT. Here, the Δ*C*_*T*_ on the *x*-axis represents each strain grown without Cm (internal control). Data points and error bars represent means ± SEM from ≥3 independent biological replicates. *craA* expression was significantly induced by Cm in WT (∆∆*C*_*T*_ = 3.80 ± .60, fold change = 13.9), Δ*adeIJ* (∆∆*C*_*T*_ = 3.43 ± .45, fold change = 10.8), and Δ*adeAB* (∆∆*C*_*T*_ = 3.98 ± .44, fold change = 15.8). Statistical significance was determined using Tukey’s honest significant differences test (*P* < 0.001).

To evaluate the regulatory impact of efflux pump gene deletions on the expression of other efflux pump genes and on *ompA*, we measured the expression levels of *adeB*, *adeG*, *adeJ*, *craA*, and *ompA* across all knockout strains. Surprisingly, deletion of *adeAB*, *adeIJ*, or *craA* did not significantly alter the expression of other efflux pump genes or *ompA* relative to the wild type, as indicated by ∆∆*C*_*T*_ values near zero (Fig. 2A through C). Notably, high ∆*C*_*T*_ values for *ompA* indicate their high basal expression (Fig. 2A through C), independent of chloramphenicol exposure.

Given the uncertain status of constitutive *craA* expression in RND efflux pump knockout strains (14, 55), we quantified *craA* transcript levels in the wild-type, Δ*adeAB*, and Δ*adeIJ* strains. Under untreated conditions, *craA* expression remained relatively low (wild type: ∆*C*_*T*_ = –4.77 ± 0.26; Δ*adeIJ*: ∆*C*_*T*_ = –4.98 ± 0.30; Δ*adeAB*: ∆*C*_*T*_ = –3.98 ± 0.34) (Fig. 2D through G). Consistent with previous observations (14), *craA* expression was significantly induced by chloramphenicol in all strains except Δ*craA* mutant (wild type: ∆∆*C*_*T*_ = 3.80 ± .60, *P* < 0.001; Δ*adeIJ*: ∆∆*C*_*T*_ = 3.43 ± .45, *P* < 0.001; Δ*adeAB*: ∆∆*C*_*T*_ = 3.98 ± .44, *P* < 0.001) (Fig. 2D through G), demonstrating that *craA* is not constitutively expressed but inducible under chloramphenicol stress. Conversely, expression of RND efflux pumps (*adeB*, *adeG*, and *adeJ*) remained unchanged. Although *adeG* was expressed at a lower level relative to *adeB* and *adeJ*, its role appears limited. Additionally, *ompA* expression was slightly reduced in the Δ*adeIJ* strain upon chloramphenicol treatment (∆∆*C*_*T*_ = –1.08 ± 0.47, *P* = 0.339), although this difference was not statistically significant. These data collectively suggest that CraA is the principal efflux determinant responsible for chloramphenicol resistance in *A. baumannii* ATCC19606.

### Global transcriptomic responses in efflux pump knockout mutants

**Fig 3:**
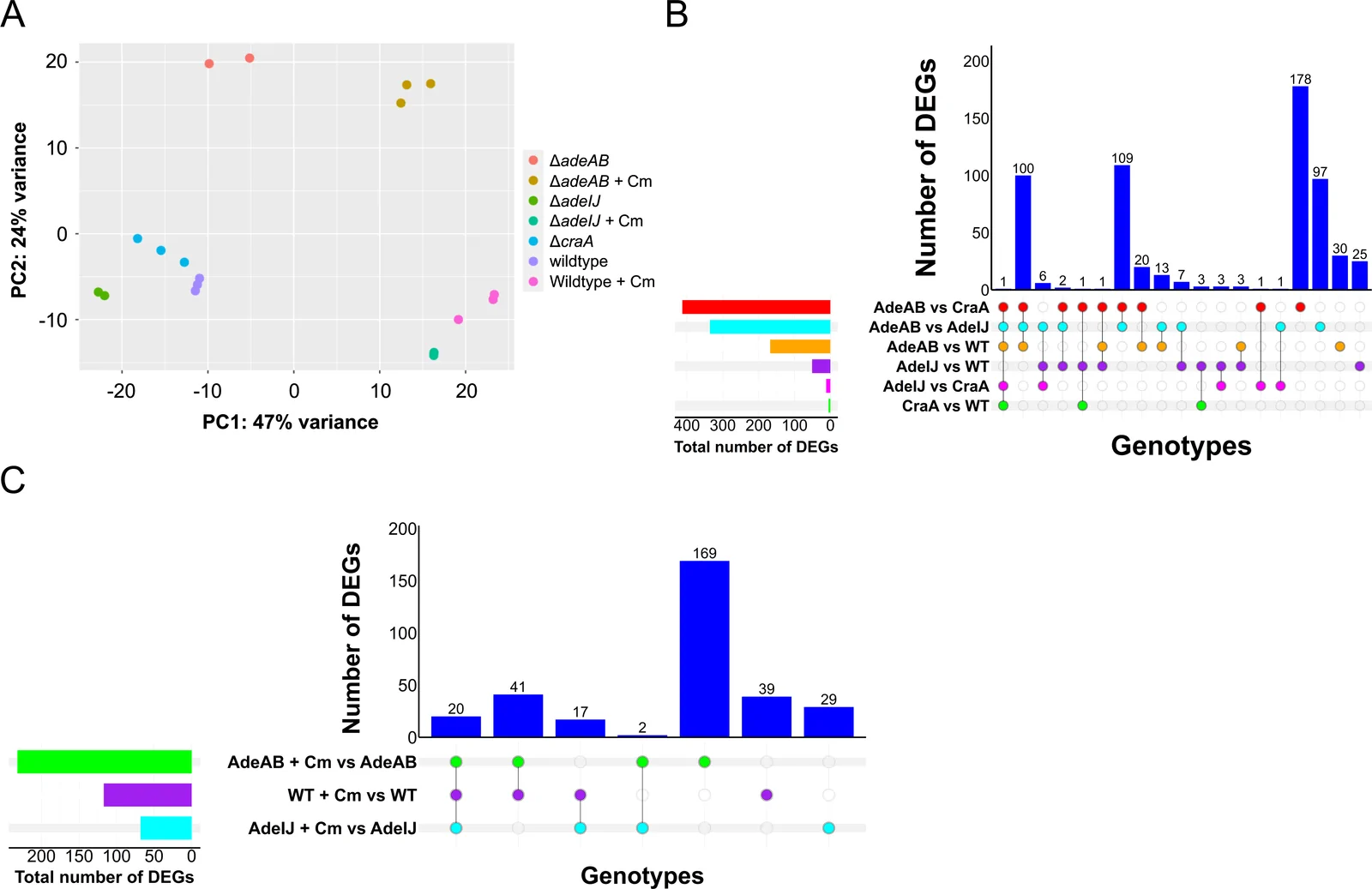
Gene expression analysis of *A. baumannii* ATCC19606 wild-type and efflux pump knockout strains. (**A**) Principal component analysis (PCA) of the normalized RNA-seq data of *A. baumannii* ATCC19606 strains in response to chloramphenicol (Cm). UpSet plots show upregulated and downregulated significant genes (log₂FC > 1.2 or < −1.2; adjusted *P*-values < 0.05) of (**B**) *A. baumannii* ATCC19606 wild type in response to RND knockout strains and (**C**) *A. baumannii* ATCC19606 strains in response to chloramphenicol.

To investigate the physiological roles of major efflux systems in *A. baumannii* ATCC19606 under basal conditions, we performed RNA sequencing of wild-type and isogenic Δ*adeAB*, Δ*adeIJ*, and Δ*craA* knockout strains grown in LB. Principal component analysis (PCA) demonstrated tight clustering among biological replicates and distinct separation between wild-type and mutant strains (Fig. 3A), supporting high-quality transcriptomic data and clear genotype-dependent expression patterns. Among the mutants, Δ*adeAB* displayed the most pronounced global transcriptional changes, with 413, 336, and 168 differentially expressed genes (DEGs) relative to Δ*craA*, Δ*adeIJ*, and wild type, respectively (|log₂FC| > 1.2, adjusted *P*-value < 0.05) (Fig. 3; Data set S1). In comparison, Δ*adeIJ* and Δ*craA* strains exhibited significantly fewer DEGs (51 and 5 genes, respectively; Fig. 3B; Data set S1). These results suggest that AdeAB may play a broader role in cellular homeostasis beyond its function in drug efflux.

### Efflux pump inactivation induces distinct pathway level adaptations

**Fig 4:**
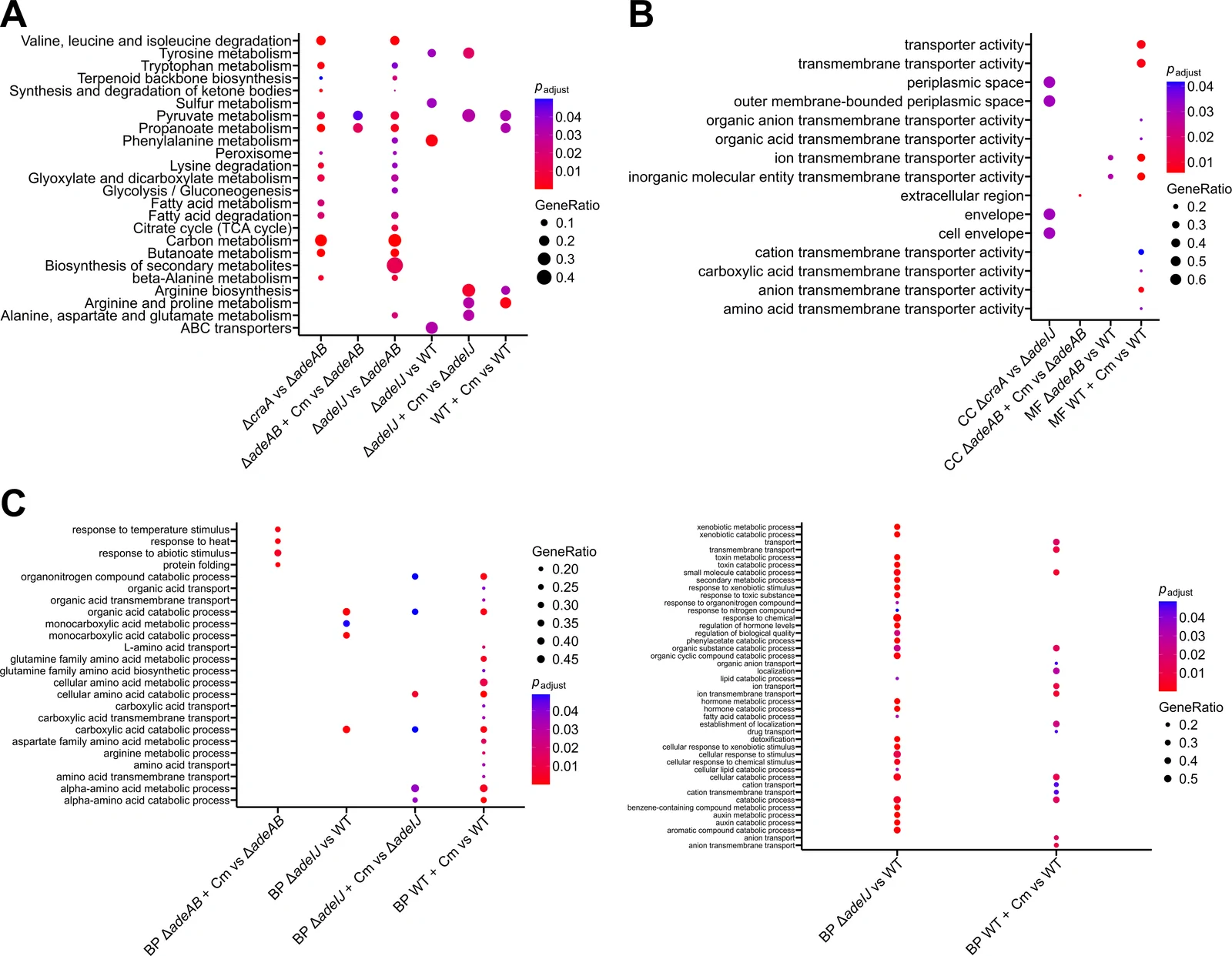
Enrichment analysis of differentially expressed genes with the (**A**) Kyoto Encyclopedia of Genes and Genomes (KEGG) pathway, (**B**) Gene Ontology (GO) categories of molecular function (MF) and cellular component (CC), and (**C**) GO category of biological process (BP).

**Fig 5:**
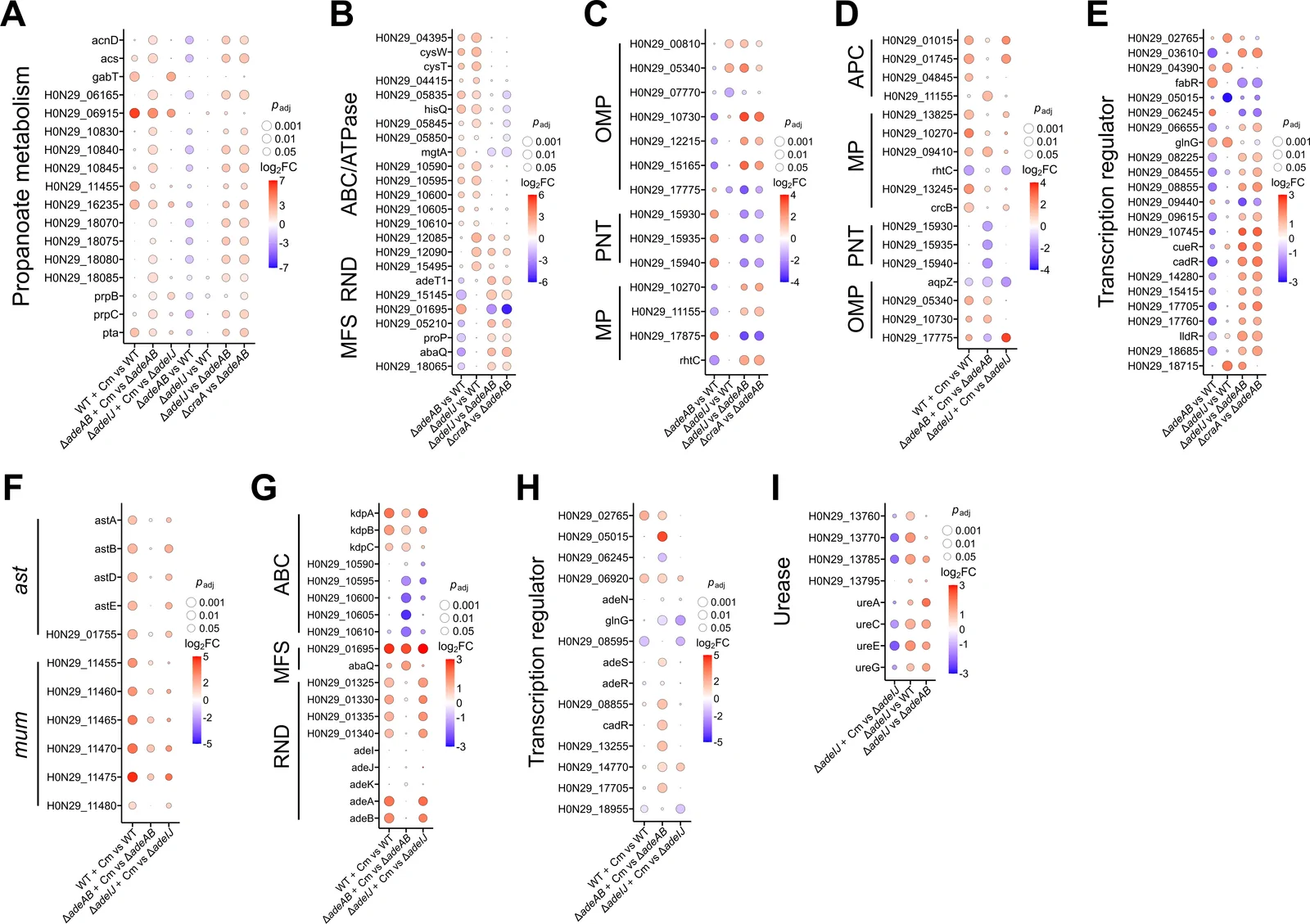
Heatmap clustering of genes associated with (**A**) propanoate metabolism; (**B and C**) efflux pumps, proton translocating transhydrogenase, and membrane proteins modulated by RND knockout; (**D**) membrane proteins responsive to chloramphenicol stress; (**E**) transcriptional regulators affected by RND knockout; (**F–H**) *ast* and *mum* operons, transporters, and transcriptional regulators modulated by chloramphenicol stress; and (**I**) urease operon. Red and blue dots represent up- and downregulated genes, respectively. Dot size corresponds to statistical significance, with larger dots indicating lower adjusted *P*-values and smaller dots indicating larger adjusted *P*-values. Please note that H0N29_01695 corresponds to *craA*.

KEGG and GO enrichment analyses revealed that inactivation of RND efflux pumps, particularly AdeAB and AdeIJ, triggered distinct physiological responses in *A. baumannii* ATCC19606 (Fig. 4; Tables S2 and S3). Compared to Δ*adeIJ*, the Δ*adeAB* mutant showed significant enrichment of 18 KEGG pathways, while only four were enriched in the wild-type strain, and none were significantly enriched in comparisons involving Δ*craA* (Fig. 4A; Table S2). Notably, 13 KEGG pathways, including those related to amino acid metabolism, fatty acid metabolism, and central carbon metabolism, were commonly perturbed in Δ*adeAB* and Δ*adeIJ*, as well as in Δ*adeAB* and Δ*craA*, indicating partially overlapping regulatory effects (Fig. 4A; Table S2). Additionally, in the Δ*adeAB*, propanoate metabolism was significantly downregulated under a less stringent threshold (adjusted *P*-value < 0.1) (Fig. 5A; Table S4).

The Δ*adeIJ* strain exhibited 35 significantly enriched GO terms, primarily associated with responses to chemical stimuli, detoxification, and organic acid metabolism (Fig. 4C; Table S3). Key upregulated genes included those encoding ABC transporters, heavy metal efflux systems (e.g., *copB*, copper ATPases), and phenylacetate degradation enzymes (Fig. 5B; Table S3), suggesting metabolic burden and metal ion accumulation upon loss of AdeIJK. In contrast, GO analysis identified only two enriched terms in Δ*adeAB*, both related to transmembrane transporter activity (Fig. 4B; Table S3). Among the upregulated genes were the multidrug efflux transporter *craA*, *hisQ*, *mgtA*, and a putative NAD(P)+ transhydrogenase operon (H0N29_15930–15940) (Fig. 5B and C; Table S3), the latter of which was notably repressed under chloramphenicol stress (Fig. 5C and D; Data set S1).

Interestingly, when a less stringent significance threshold (adjusted *P*-value < 0.1) was applied, additional DEGs categories were uniquely enriched in Δ*adeAB*, including pathways involved in RNA processing and iron–sulfur (Fe–S) cluster biogenesis (Tables S4 and S5; Data set S1). Notably, *iscU* and *iscA*, key components of the Fe–S cluster assembly machinery, were upregulated, along with several RNA-binding and processing genes (Table S5; Data set S1). These transcriptional changes may indicate that loss of AdeAB is associated with a cellular stress response linked to redox imbalance and RNA metabolism. Furthermore, unlike Δ*adeIJ* and Δ*craA*, the Δ*adeAB* strain also exhibited substantial downregulation of transcriptional regulators (Fig. 5E), which may indicate alterations in regulatory network activity. Together, these findings point to functional differences among RND efflux pumps and suggest that, while AdeIJK may play a broad-spectrum detoxification and membrane remodeling, AdeAB could have roles extending beyond drug efflux, potentially influencing metabolic and regulatory homeostasis.

### Chloramphenicol induces distinct metabolic and transcriptional changes in wild-type and RND efflux pump knockout strains

To characterize efflux-independent adaptation to chloramphenicol, we exposed wild-type and mutant strains to sub-MIC of chloramphenicol and performed comparative transcriptomic profiling. PCA confirmed sample reproducibility and revealed separation between treated and untreated groups for each genotype (Fig. 3A). Chloramphenicol treatment elicited significant transcriptional shifts, with the extent of the response varying by strains, 232 DEGs in Δ*adeAB*, 117 DEGs in wild type, and 68 DEGs in Δ*adeIJ* (Fig. 3C; Data set S1). To elucidate the metabolic adaptations of *A. baumannii* ATCC19606 under chloramphenicol exposure, we analyzed DEGs in the wild-type, Δ*adeAB*, and Δ*adeIJ* strains for KEGG pathway and Gene Ontology (GO) term enrichment (Fig. 4; Tables S2 and S3). Six KEGG pathways were significantly enriched in response to chloramphenicol across all strains (Fig. 4A; Table S2). Notably, pyruvate metabolism (map00620; adjusted *P*-value < 0.05) was consistently enriched, involving transcripts H0N29_06915 and H0N29_06930, both markedly upregulated (log₂FC > 3.5) (Data set S1). These genes are putatively associated with ethanol metabolism, suggesting a chloramphenicol response independent of RND-type efflux mechanisms.

In addition to pyruvate metabolism, arginine/proline metabolism, and arginine biosynthesis pathways were conserved between the wild-type and Δ*adeIJ* strains (Fig. 4A). These included the upregulated *astB* and H0N29_06915 transcripts as well as H0N29_10275 (Fig. 4A and 5F; Table S2). Notably, the arginine succinyltransferase (*ast*) operon, comprising *astA*, *astB*, *astD*, *astE*, and H0N29_01755, was upregulated in the wild-type strain (log₂FC > 1.6, adjusted *P*-value < 0.05; Fig. 5F; Data set S1). In the Δ*adeIJ* mutant, only *astB* was significantly upregulated, although the other operon members showed similar expression trends without statistical significance (Fig. 5F; Table S5; Data set S1). Enrichment of the arginine biosynthesis pathway under chloramphenicol stress is intriguing, as this pathway has been primarily linked to bactericidal antibiotic lethality rather than bacteriostatic responses (56). In contrast, transcripts involved in the propanoate metabolism pathway, including H0N29_06915 and H0N29_16235, were enriched in both Δ*adeAB* and the wild-type strain (Fig. 4A and 5A; Table S2). Interestingly, tyrosine metabolism and alanine/aspartate/glutamate metabolism pathways were uniquely enriched in the Δ*adeIJ* strain (Fig. 4A; Table S2).

GO term analysis revealed that DEGs from the wild-type strain were enriched in categories associated with transport, amino acid metabolism, and organic acid metabolism (Fig. 4B and C; Table S3). Δ*adeAB* DEGs, however, were enriched in stress-associated processes, such as environmental response, protein folding, and extracellular region localization (Fig. 4B and C; Table S3). These included protease- and chaperone-associated genes (e.g., H0N29_03910, *groL*, *grpE*, *dnaK*, *clpB*, *htpG*, H0N29_01635, and *lon*), as well as *uvrB* and several hypothetical proteins (Table S3). Notably, cold-shock proteins H0N29_06030 and H0N29_12010 were downregulated in Δ*adeAB* (Data set S1), although they were not annotated within any GO category. The robust upregulation of proteostasis-related genes in Δ*adeAB* under sub-inhibitory chloramphenicol exposure is particularly notable, as such responses are more commonly associated with bactericidal stress (20, 24). In contrast, DEGs in Δ*adeIJ* were primarily enriched in amino acid and organic acid metabolism pathways (Fig. 4B and C; Table S3).

### Transporter and efflux pump modulation under chloramphenicol stress

Among chloramphenicol-exposed strains, transcripts encoding the multidrug efflux pumps *adeA*, *adeB*, and *craA* (H0N29_01695) were among the most abundant, except in the Δ*adeAB* strain where *adeAB* is deleted (Fig. 5G; Data set S1). The strong expression of *craA* aligns with its established role as the principal chloramphenicol resistance determinant in ATCC19606 (Fig. 1 and 2B; Table 1; Fig. 4B; Table S3) (14, 28). Intriguingly, *craA* was also upregulated even in the absence of chloramphenicol in the Δ*adeAB* background (Fig. 5B; Data set S1). Furthermore, induction of *adeA* and *adeB* under chloramphenicol exposure appeared to coincide with increased *craA* expression (Fig. 5G; Data set S1), suggesting possible coordinated or complementary efflux activity among these transporters (28). In contrast, *adeI* and *adeJ* expression remained largely unchanged across conditions (Fig. 5G; Data set S1).

Transcripts encoding putative heavy metal resistance efflux systems (CusA/CzcA family; H0N29_01325–H0N29_01340) were upregulated in both Δ*adeIJ* and wild-type strains during chloramphenicol stress (Fig. 5G; Data set S1). By contrast, genes such as *adeT1* and H0N29_15145, which encode components of a tripartite efflux system, were differentially expressed in Δ*adeAB*, but were not induced by chloramphenicol in any strain (Fig. 5B and G; Data set S1). The MFS transporter *abaQ* was significantly upregulated in Δ*adeAB* under chloramphenicol (Fig. 5G; Data set S1). In Δ*adeAB*, *abaQ* upregulation coincided with elevated expression of the adjacent genes H0N29_08175 and H0N29_08180 encoding fumarylacetoacetate hydrolase and aldehyde dehydrogenase, respectively (Data set S1), consistent with their polycistronic organization (57).

Beyond classical efflux systems, multiple transporters were differentially expressed. In the wild-type strain, GO enrichment for “transmembrane transporter activity” (GO:0022857) highlighted genes implicated in amino acid or ion transport, were significantly enriched (Fig. 3B; Table S3). Chloramphenicol exposure also induced the potassium transporter complex KdpABC and amino acid permease H0N29_01015 while repressing the water channel *aqpZ* in Δ*adeIJ* (Fig. 5D and G; Table S3). These findings are accorded with prior reports linking *kdpA* to monovalent cation-mediated antibiotic tolerance in *A. baumannii* (58). Additionally, the amino acid ABC transporter operon H0N29_10590–H0N29_10610 (excluding H0N29_10590) was significantly downregulated in Δ*adeAB* (Fig. 5G).

### Limited impact of chloramphenicol on outer membrane protein gene expression

The inherently low outer membrane permeability of *A. baumannii* contributes to its intrinsic resistance (3, 4). Despite previous studies reporting that inactivation of outer membrane proteins (OMP) genes such as *abuO* and *ompA* increases chloramphenicol susceptibility (59, 60), our transcriptomic analysis showed that chloramphenicol exposure and efflux pump deletions did not substantially alter the expression of major OMP genes, including *abuO*, *carO*, *ompA*, *ompW*, and *omp33–36* (Fig. 5C and D; Data set S1). Several TonB-dependent receptors, however, showed strain-specific regulation. For example, H0N29_07770 and H0N29_00810 were specifically modulated in Δ*adeIJ*, while H0N29_12215 and H0N29_10730 were downregulated in Δ*adeAB* (Fig. 5C). Additionally, H0N29_05340 (putative OmpW family protein) and H0N29_17775 (putative TonB-dependent copper receptor) were differentially expressed in wild-type and Δ*adeIJ* strains under chloramphenicol stress, respectively (Fig. 5D). As chloramphenicol uptake depends on porin (6, 7), our findings suggest that resistance is not driven by transcriptional changes in OMP abundance, but rather that OMP regulation during antibiotic stress likely occurs at the post-transcriptional level (58, 61).

### Transcriptional regulators and additional stress response genes

Given the established roles of transcriptional regulators such as AdeRS and AdeN in controlling AdeABC and AdeIJK responses to antibiotics (16, 17), we examined regulator expression during chloramphenicol stress. Unexpectedly, chloramphenicol did not broadly induce canonical efflux regulators, including *adeRS* and *adeN* (Fig. 5H; Data set S1). Instead, regulatory changes were selective. The TetR/AcrR-type regulator H0N29_05015, homologous to A1S_2396 in ATCC17978, was significantly downregulated in Δ*craA* and Δ*adeIJ* strains (Fig. 5E; Data set S1). Another regulator, H0N29_08595, was repressed in the wild-type and Δ*adeIJ* strains under chloramphenicol (Fig. 5H). In contrast, although several regulators were reduced in Δ*adeAB*, chloramphenicol produced minimal additional transcriptional effects in this mutant (Fig. 5E and H).

Beyond efflux mechanisms, we observed conserved prophage gene enrichment within the H0N29_05405–H0N29_05795 cluster across all strains (Table S5). This supports the emerging view that prophages may contribute to antibiotic stress tolerance and environmental adaptation in *A. baumannii* (62, 63). Intriguingly, chloramphenicol exposure led to repression of the type VI secretion system (T6SS) only in the Δ*adeAB* strain (Table S5). In contrast, the Δ*adeAB* strain showed upregulation of T6SS genes in the absence of the antibiotic (Table S5). The manganese and urea metabolism (*mum*) operon, important for metal homeostasis and virulence (64), was strongly upregulated in the wild-type strain in response to chloramphenicol (Fig. 5F), except for its LysR-type regulator H0N29_11480. Interestingly, this operon is downregulated under other stressors such as ethanol and hydrogen peroxide (65, 66), suggesting chloramphenicol-specific regulation. However, deletion of *mumT* did not impact chloramphenicol susceptibility, suggesting that its induction contributes to adaptation rather than providing intrinsic resistance (Fig. S2).

### Oxidative and metal-mediated stress responses in *A. baumannii* efflux pump mutants

Intracellular reactive oxygen species (ROS) generated via Fenton chemistry are a hallmark of oxidative stress (67). Transcriptomic analysis suggested that RND knockout strains may experience elevated oxidative stress, reflected by the upregulation of heavy metal efflux systems and iron-sulfur cluster biogenesis genes (Fig. 5B and G; Table S5; Data set S1). To further validate this observation, we phenotypically assessed the strains under hydrogen peroxide (H_2_O_2_) and sodium nitroprusside (SNP), and quantified intracellular ROS using 2′,7′-dichlorodihydrofluorescein fluorescence.

**Fig 6:**
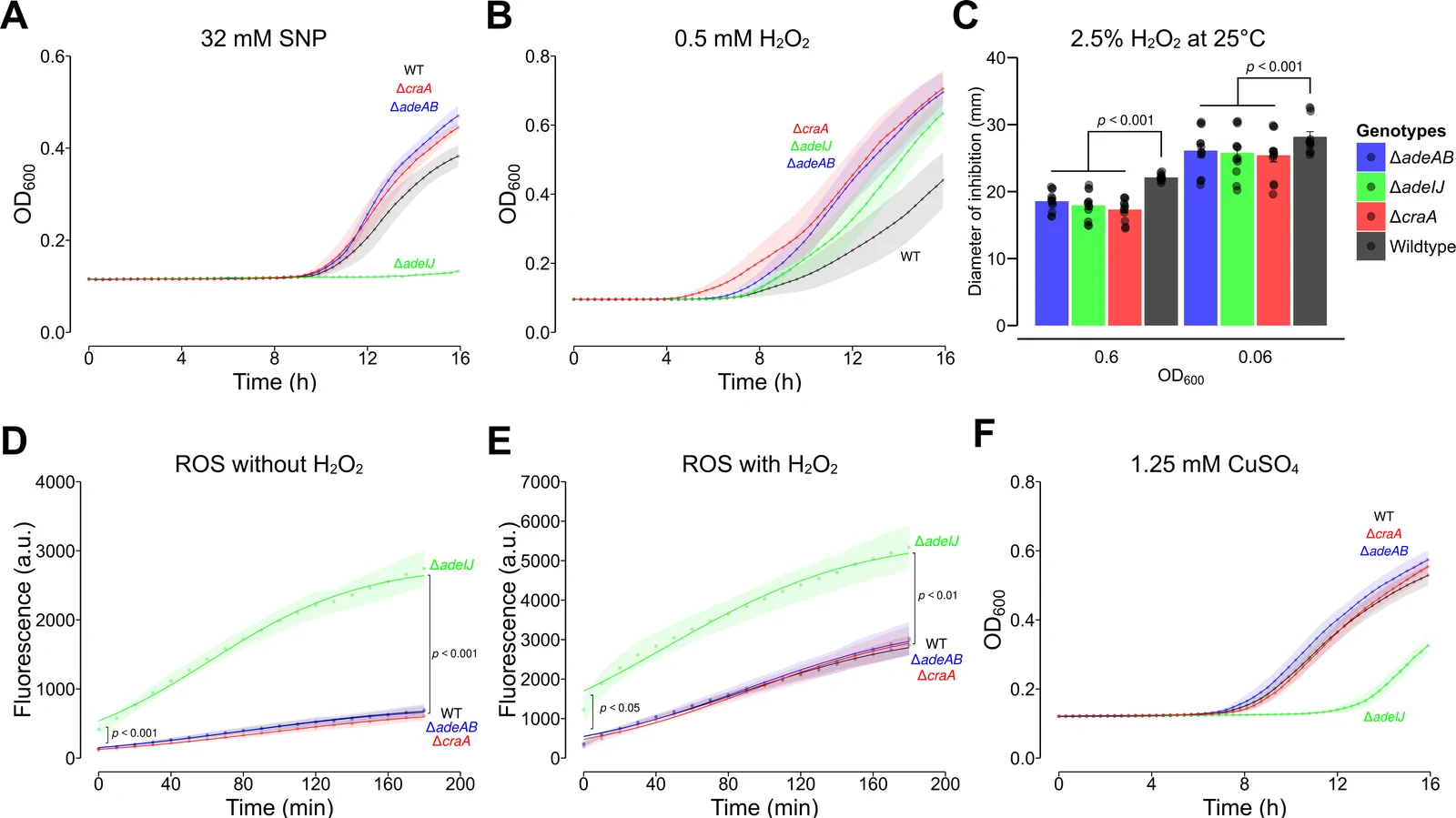
Oxidative and metal stress responses in *A. baumannii* wild-type (WT) and knockout strains. Data represent means ± SEM from at least three independent biological replicates. (**A and B**) Growth curves of *A. baumannii* strains cultured in LB supplemented with (**A**) 32 mM SNP or (**B**) 0.5 mM H_2_O_2_. (**C**) Growth inhibition zones on LB agar plates containing 2.5% H_2_O_2_. Statistical significance was determined using general linear hypothesis testing followed by Tukey’s multiple comparison test. (**D and E**) Intracellular ROS levels quantified by 2′,7′-dichlorodihydrofluorescein fluorescence in the (**D**) absence or (**E**) presence of H_2_O_2_. Statistical comparisons among strains were performed using the Tukey’s HSD test. (**F**) Growth curves of *A. baumannii* variants in LB medium supplemented with 1.25 mM CuSO_4_.

Despite being able to grow in LB supplemented with 16 mM SNP, the Δ*adeIJ* mutant consistently exhibited a prolonged lag phase, reduced μ_max_, and increased doubling time (*T*_*d*_), although these differences were not statistically significant (Fig. S3A through D). In contrast, Δ*craA* displayed a shorter lag phase and higher OD_max_, although the latter did not reach statistically significant levels (Fig. S3A and B). Under 32 mM SNP, however, Δ*adeIJ* showed pronounced growth inhibition (Fig. 6A). Strikingly, inactivation of *craA* or *adeAB* conferred enhanced tolerance to higher SNP concentrations, reflected by increased μ_max_ and decreased *T*_*d*_ relative to the wild type (Fig. 6A; Fig. S3A, C, and D).

In contrast, none of the strains grew in LB containing 1 mM H_2_O_2_, and the wild-type strain was particularly sensitive at 0.5 mM H_2_O_2_ (Fig. 6B). Under exposure to 0.5 mM H_2_O_2_, all strains exhibited comparable μ_max_ values (0.47–0.59 h^−1^), although the wild type showed a slightly lower μ_max_ than the Δ*craA* and Δ*adeIJ* mutants (Fig. S4A). However, the wild type exhibited a markedly prolonged lag phase, delayed *T*_μmax_, reduced OD_max_, and a smaller AUC compared to the RND and *craA* knockout strains (Fig. S4B through E). Intriguingly, the Δ*craA* and Δ*adeAB* mutants demonstrated enhanced tolerance to H_2_O_2_, evidenced by a significantly shorter lag phase, increased *T*_μmax_, enhanced OD_max_, and larger AUC (Fig. 6B; Fig. S4). The Δ*adeIJ* mutant, however, showed an intermediate response between the wild-type and the Δ*craA*/Δ*adeAB* mutants, although these differences were not statistically significant (Fig. 6B; Fig. S4). In agreement with these results, the wildtype exhibited greater growth inhibition on H_2_O_2_-containing solid medium at both 25°C and 37°C (Fig. 6C; Fig. S5). Consistent with transcriptomic observations, Δ*craA* displayed ROS levels comparable to the wild type (Fig. 6C), suggesting that *craA* inactivation does not substantially alter intracellular redox balance. Unexpectedly, despite the induction of oxidative stress-related genes (Fig. 4B and C; Table S3), Δ*adeAB* maintained ROS levels similar to the wild type (Fig. 6C). In contrast, Δ*adeIJ* exhibited significantly elevated basal ROS relative to all other strains after 30 min of incubation with the fluorescent probe (Fig. 6C), and ROS levels continued to rise over 180 min, indicating a loss of redox homeostasis upon *adeIJ* deletion. Upon H_2_O_2_ challenge, ROS levels increased in all strains (Fig. 6D), but Δ*adeIJ* showed a disproportionately higher accumulation (Fig. 6D), underscoring its heightened oxidative stress sensitivity. Collectively, these findings suggest that disruption of RND and MFS efflux pumps may be accompanied by compensatory physiological or regulatory adaptations that could influence oxidative stress tolerance in *A. baumannii*.

Since both a putative CusA/CzcA family efflux system (H0N29_01325–H0N29_01340) and the *mum* operon involved in metal homeostasis (64, 68) were upregulated in Δ*adeIJ* and wild type under chloramphenicol stress, respectively (Fig. 5F and G), we next assessed the growth kinetics of all strains under copper and manganese stress in the absence of antibiotics. Unexpectedly, none of the strains grew in LB supplemented with 2.5 mM Cu^2+^ (Table 1), and the Δ*adeIJ* strain exhibited pronounced growth inhibition even at 1.25 mM Cu^2+^, despite exhibiting comparable μ_max_ values (Δ*adeIJ*: 0.30 h^−1^; others: 0.28–0.31 h^−1^) (Fig. 6F; Fig. S4A). Notably, Δ*adeIJ* displayed a prolonged lag phase, delayed *T*_μmax_, reduced OD_max_, and smaller AUC relative to the wild type and other mutants (Fig. S4B through E). In contrast, the growth of RND and *craA* knockout strains was unaffected by Mn^2+^ supplementation (Fig. S6). Together, the upregulation of copper efflux components such as *copB* and copper ATPases, along with the increased copper sensitivity of Δ*adeIJ* (Fig. 5B and G and 6E; Data set S1), suggests that loss of AdeIJ may be associated with alterations in copper homeostasis and altered cellular physiology linked to disrupted metal ion regulation.

## DISCUSSION

Efflux pumps are central determinants of intrinsic and acquired antibiotic resistance in *A. baumannii*, acting in concert with low membrane permeability and antibiotic-modifying enzymes (3, 4). Under standard growth conditions, deletion of the RND systems (Δ*adeIJ*, Δ*adeAB*) or the MFS CraA in ATCC19606 caused only minimal transcriptomic perturbations, consistent with in strain AB5075 (27). However, both the response to chloramphenicol and the regulatory response to RND knockout under non-stress conditions were strain-specific, reflecting the consequences of functional redundancy among efflux systems. Whereas AB5075 showed induction of biofilm formation, efflux pumps, fatty acid, iron, and carbon metabolism genes (27), Δ*adeIJ* in ATCC19606 primarily upregulated phenylacetate catabolism and ABC transporters (Fig. 4A and C and 5B; Tables S2 and S3). In contrast, Δ*adeAB* showed pronounced transcriptional remodeling, marked by upregulation of ribosomal proteins, transporters including CraA, and [Fe-S] cluster biogenesis, together with repression of propanoate metabolism (Fig. 4B and 5A to C; Tables S2 through S5). The Δ*adeAB* mutant also exhibited increased tolerance to H_2_O_2_, suggesting that induction of stress-related pathways following *adeAB* deletion may confer cross-protection against oxidative stress. Given the role of [Fe-S] cluster maintenance in oxidative stress resistance (69), these transcriptional changes may represent compensatory mechanisms that help mitigate redox imbalance, consistent with previous reports linking RND pumps to oxidative stress tolerance (70, 71).

**Fig 7:**
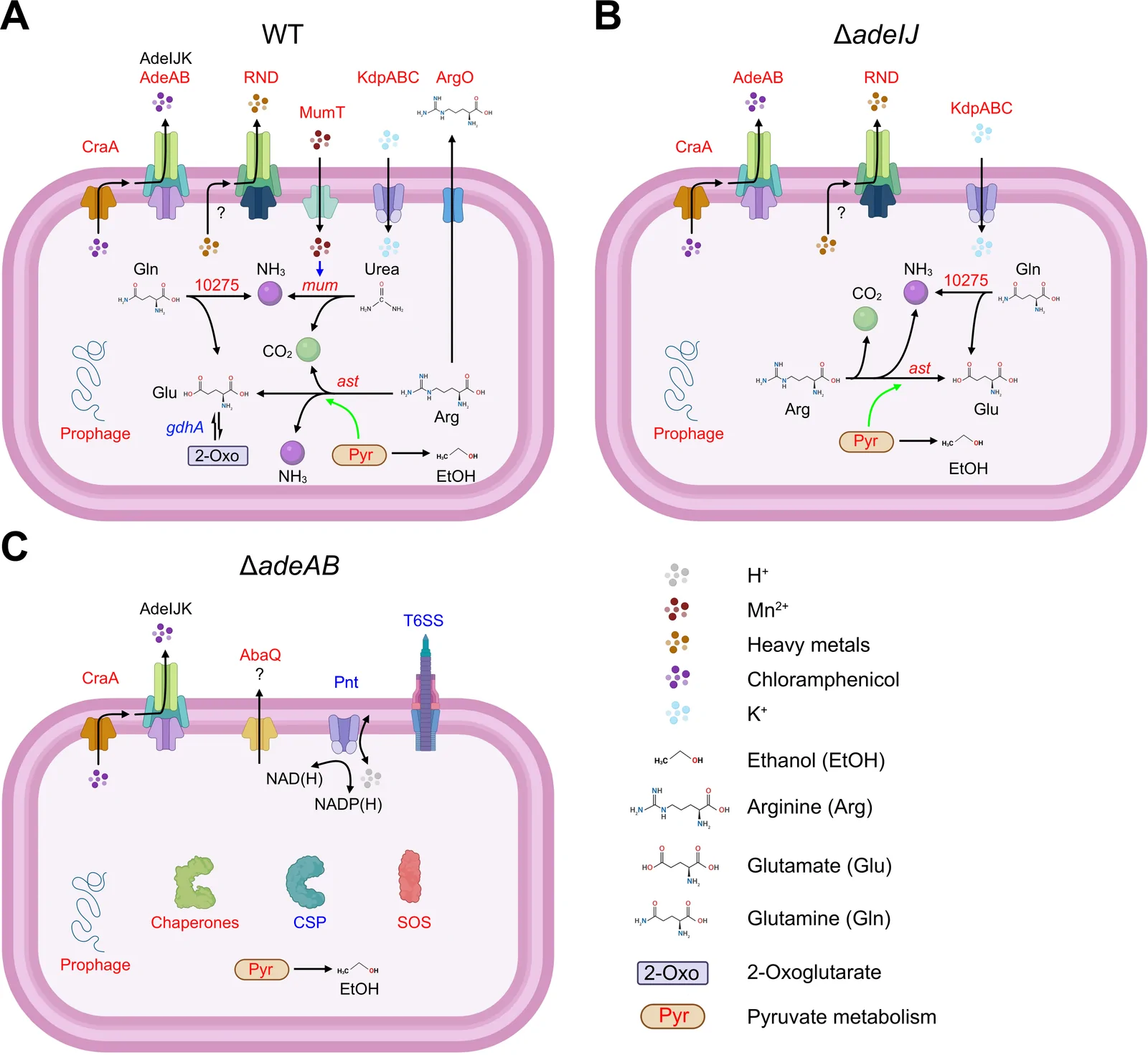
Schematic representation of the chloramphenicol resistance determinants and chloramphenicol-induced stress response in (**A**) wild-type, (**B**) Δ*adeIJ*, and (**C**) Δ*adeAB* strains of *A. baumannii* ATCC19606. CraA functions as the primary efflux determinant conferring resistance to chloramphenicol, whereas AdeABC and AdeIJK contribute auxiliary roles in chloramphenicol resistance. The curved arrows on CraA indicate transport of chloramphenicol from the cytoplasm to periplasm, where RND efflux pumps (AdeABC and/or AdeIJK) subsequently export the drug across the outer membrane. DEGs involved in the stress response are color-coded: red indicates upregulation, and blue indicates downregulation. No significant changes in *adeIJK* expression were observed in the wild-type or Δ*adeAB* strains in response to chloramphenicol, while the *adeI* and *adeJ* genes are deleted in the Δ*adeIJ* mutant. The green arrow from pyruvate metabolism indicates a potential contribution of precursors, likely via succinyl-CoA, to the AST pathway for arginine degradation. Similarly, the blue arrow from MumT suggests manganese supplementation to the *mum* operon for urea degradation. RND, a putative heavy metal resistance efflux pump (H0N29_01325–H0N29_01340); 10275, a putative type II asparaginase (H0N29_10275); *gdhA*, a putative NADP-specific glutamate dehydrogenase; ArgO, a putative arginine transporter (H0N29_13245); CSP, putative cold-shock proteins (H0N29_06030 and H0N29_12010); Pnt, a putative proton translocating transhydrogenase; *mum*, H0N29_11455–H0N29_11480. A question mark on AbaQ indicates unknown substrates, while the question mark adjacent to the putative heavy metal resistance efflux pump (RND) denotes an uncharacterized inner membrane transporter.

Upon chloramphenicol exposure, Δ*adeAB* displayed a distinct transcriptional profile, including increased expression of NAD(P)^+^ transhydrogenases, SOS responses, and protein quality control systems such as proteases and chaperones (Fig. 4A, 5D, and 7C; Tables S2 through S5). Concurrently, cold-shock proteins (Fig. 7C; Data set S1), which have been reported to influence chloramphenicol susceptibility (72), were downregulated, possibly reflecting a redistribution of stress-response resources. Repression of the type VI secretion system (T6SS) in Δ*adeAB* is consistent with observations that T6SS inactivation can be associated with enhanced antibiotic resistance (73) (Fig. 7C; Table S5), whereas its activation may increase oxidative sensitivity under Mn²^+^ limitation (46). Previous studies have also reported that T6SS-positive *A. baumannii* clinical isolates tend to exhibit higher resistance rates to several antimicrobials (74). However, whether this reflects a direct contribution of T6SS activity to antimicrobial susceptibility or broader strain-specific regulatory differences remains unclear. Given the concurrent induction of redox- and stress-related pathways in Δ*adeAB*, repression of T6SS under chloramphenicol stress may represent an additional adaptive adjustment to limit intracellular stress. Overall, these data suggest that deletion of *adeAB* triggers broad metabolic and regulatory changes that may extend beyond contribution to chloramphenicol efflux.

Under chloramphenicol stress, *adeA* and *adeB* transcripts increased in the wild-type and Δ*adeIJ* strains, yet deletion of *adeAB* did not alter MIC values, whereas Δ*craA* and Δ*adeIJ* exhibited a 32- and 4-fold MIC reduction, respectively (Table 1). Consistent with substrate-responsive mode of regulation (14), *craA* was upregulated in all mutants except Δ*craA* (Fig. 2D through G). Beyond chloramphenicol susceptibility, Δ*craA* exhibited increased tolerance to SNP and H_2_O_2_ (Fig. 6A and B; Fig. S3 and S4), suggesting a potential link to redox homeostasis. In contrast, Δ*adeIJ* accumulated higher basal and induced ROS levels, displayed greater sensitivity to nitrosative and oxidative stress, and upregulated oxidative and metal-stress-associated genes, including copper transporters, consistent with its hypersensitivity to Cu^2+^ (Fig. 5G and 6A, C, and D; Data set S1). Notably, *craA* was also upregulated in the Δ*adeAB* background even in the absence of chloramphenicol (Fig. 5B; Data set S1). This observation aligns with findings in strain ATCC17978, where inactivation of *adeFGH* led to overexpression of *adeAB* (75), and with the broader concept of a coordinated, versatile bacterial efflux network comprising RND and MFS with overlapping specificities (76, 77). Collectively, the data suggest a hierarchical efflux model in which CraA plays a primary role in chloramphenicol efflux, whereas AdeIJK may contribute to redox and metal homeostasis (Fig. 7), with potential compensatory or strain-specific and condition-specific interactions between RND systems and CraA under antibiotic stress.

Beyond efflux mechanisms, chloramphenicol exposure triggered metabolic remodeling in the Δ*adeIJ* and wild-type strains, marked by increased arginine/proline metabolism, pyruvate metabolism, and urease activity (Fig. 4A, 5I, and 7A and B; Table S2). These pathways converge on glutamate biosynthesis, generating ammonia and CO₂ (Fig. 7A and B). Although inactivation of *astA*, the first gene in the *ast* operon, did not affect chloramphenicol susceptibility (Fig. S2), induction of the AST pathway, upregulation of a putative arginine exporter (*argO*), and repression of *gdhA* suggest a likely coordinated response aimed at maintaining pH balance and alleviating antibiotic stress rather than directly mediating resistance (Fig. 5D and 7A; Table S2). Enrichment of pyruvate and propanoate metabolism further supports glutamate production via increased generation of acetyl-CoA and succinyl-CoA (Fig. 7A; Table S2). Together, these results suggest that intracellular glutamate and arginine pools may contribute to protection against oxidative and membrane stress during chloramphenicol exposure.

### Conclusion

*A. baumannii* has evolved a highly versatile metabolic and nutritional strategy, enabling it to persist in hostile environments. Our findings provide a comprehensive view of strain-specific adaptations of ATCC19606 to chloramphenicol stress, establishing CraA as the principal chloramphenicol efflux determinant while suggesting potential links between RND inactivation and oxidative as well as metal stress responses (Fig. 7). However, the regulatory response to chloramphenicol appeared to be strain-specific in ATCC19606, suggesting functional redundancy among efflux systems and highlighting the limitations of extrapolating findings from single-gene deletion transcriptomic analyses. The observed connections among efflux regulation, central metabolism, and stress physiology point to the complexity of intrinsic antibiotic resistance and highlight the importance of accounting for strain-specific variation and functional redundancy among efflux systems in *A. baumannii*. Future work incorporating combinatorial deletions, regulatory mutants, and clinically derived isolates would help clarify the hierarchy and interplay of efflux systems under antibiotic stress.

## Supplementary Material

Reviewer comments

## Data Availability

Scaffolded chromosome assemblies and putative extrachromosomal contigs have been deposited in DDBJ/ENA/GenBank under accession numbers JBWVBS000000000–JBWVBV000000000.

## SUPPLEMENTAL MATERIAL

Data set S1 msystems.00009-26-s0001.xls Differentially expressed genes in *A. baumannii* strains.

Supplemental Figures msystems.00009-26-s0002.pdf Fig. S1 to S6.

Supplemental Tables msystems.00009-26-s0003.xlsx Tables S1 to S5.

OPEN PEER REVIEW reviewer-comments.pdf An accounting of the reviewer comments and feedback.

ASM does not own the copyrights to Supplemental Material that may be linked to, or accessed through, an article. The authors have granted ASM a non-exclusive, world-wide license to publish the Supplemental Material files. Please contact the corresponding author directly for reuse.
