# Supplementary material for: Chloramphenicol stress triggers oxidative adaptation in Acinetobacter baumannii ATCC19606 devoid of RND efflux pumps AdeAB or AdeIJ
Source: mSystems. 2026 Sep 3;11(9):e00009-26. doi: 10.1128/msystems.00009-26 (PMC13595967; doi:10.1128/msystems.00009-26)

Chloramphenicol Stress Triggers Oxidative Adaptation in *Acinetobacter baumannii* Devoid of RND efflux pumps AdeAB or AdeIJ

Wuen Ee Foong[1,2,3,§], Xinxin Xiang[2], Wenjun He[2], Xuan Yan[1], Jiabin Huang[4,*,§], Klaas M. Pos[5], Heng-Keat Tam[1,2,3,*]

[1]Department of Medical Microbiology, Hunan Provincial Key Laboratory for Special Pathogens Prevention and Control, Hengyang Medical School, University of South China, Hengyang, Hunan 421001, China.

[2]Department of Biochemistry and Molecular Biology, Hengyang Medical School, University of South China, Hengyang, Hunan 421001, China.

[3]National Health Commission Key Laboratory of Birth Defect Research and Prevention, Hunan Provincial Maternal and Child Health Care Hospital, Changsha, Hunan 410008, China.

[4]Institute of Medical Microbiology, Virology and Hygiene, University Medical Center Hamburg-Eppendorf, Martinistraße 52, 20246 Hamburg, Germany.

[5]Institute of Biochemistry, Goethe-University Frankfurt, Max-von-Laue-Str. 9, D-60438 Frankfurt am Main, Germany.

[§]Shared first author

*Correspondence: Jiabin Huang (j.huang@uke.de), Heng-Keat Tam (tamhk60@hotmail.com)

Supplementary Fig. 1. Drug susceptibility and ethidium efflux activity of *A. baumannii* wildtype and efflux pump knockout strains (Δ*adeAB*, Δ*adeIJ*, and Δ*craA*). (A) Half maximal inhibitory concentration (IC_{50}) of chloramphenicol against *A. baumannii* wildtype and efflux pump knockout strains. Each dot represents an individual biological replicates. (B-C) Ethidium efflux by ethidium-loaded *A. baumannii* wildtype and efflux pump knockout strains monitored fluorometrically following cellular re-energization with glucose or under non-energized conditions (No). (B) Efflux curves fitted with a sigmoid function with 95% confidence intervals (3 biological replicates, each with >4 technical replicates). (C) Ethidium efflux rate expressed as $t_{\text{efflux-50\%}}$, defined as the time required for cells to remove half of the accumulated ethidium (relative fluorescence = 0.5), either in the presence of glucose or in its absence (No). The parameter H (log-transformed $t_{\text{efflux-50\%}}$) was obtained from the sigmoid fit and converted to $t_{\text{efflux-50\%}}$ by exponentiation, $f(H) = e^{H}$. Statistical comparisons between the WT and knockout strains were performed using Tukey's multiple comparison test, $^{\#}$ indicates $P < 0.01$ (Δ*adeIJ* vs WT), $^{\$}$ indicates $P < 0.001$ (Δ*craA* vs other strains).

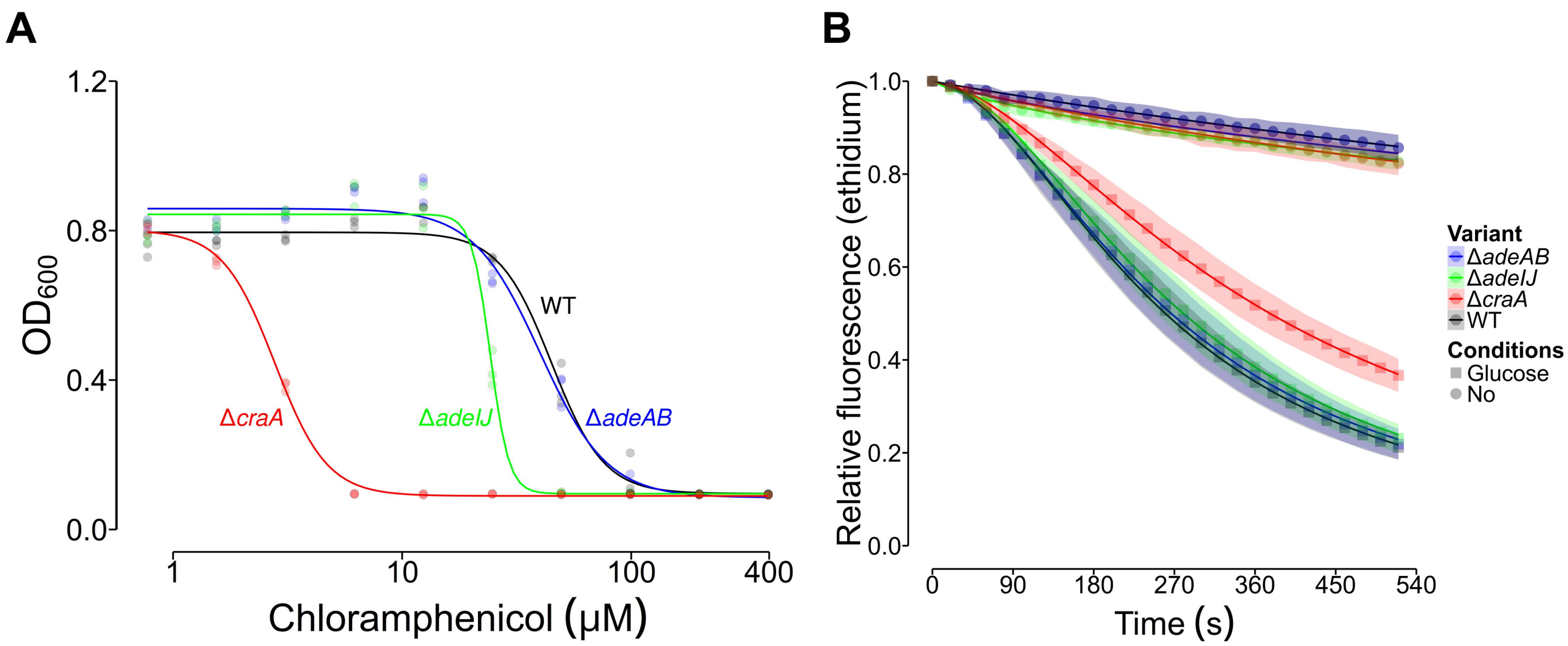

C

**Mutants**	***H***	$t_{efflux\text{-}50\%}$
Wildtype (WT)	5.54 ± 0.06	254.68
Δ*adeAB*	5.56 ± 0.06	259.82
Δ*adeIJ*	5.61 ± 0.06 #	273.14
Δ*craA*	5.88 ± 0.06 $	357.81

Supplementary Fig. 2. Drug resistance profiles of *A. baumannii* WT and knockout strains. Serially diluted overnight cultures of these strains were spotted on LB agar plates supplemented with chloramphenicol. Experiments were conducted at least three times and the result shown is representative.

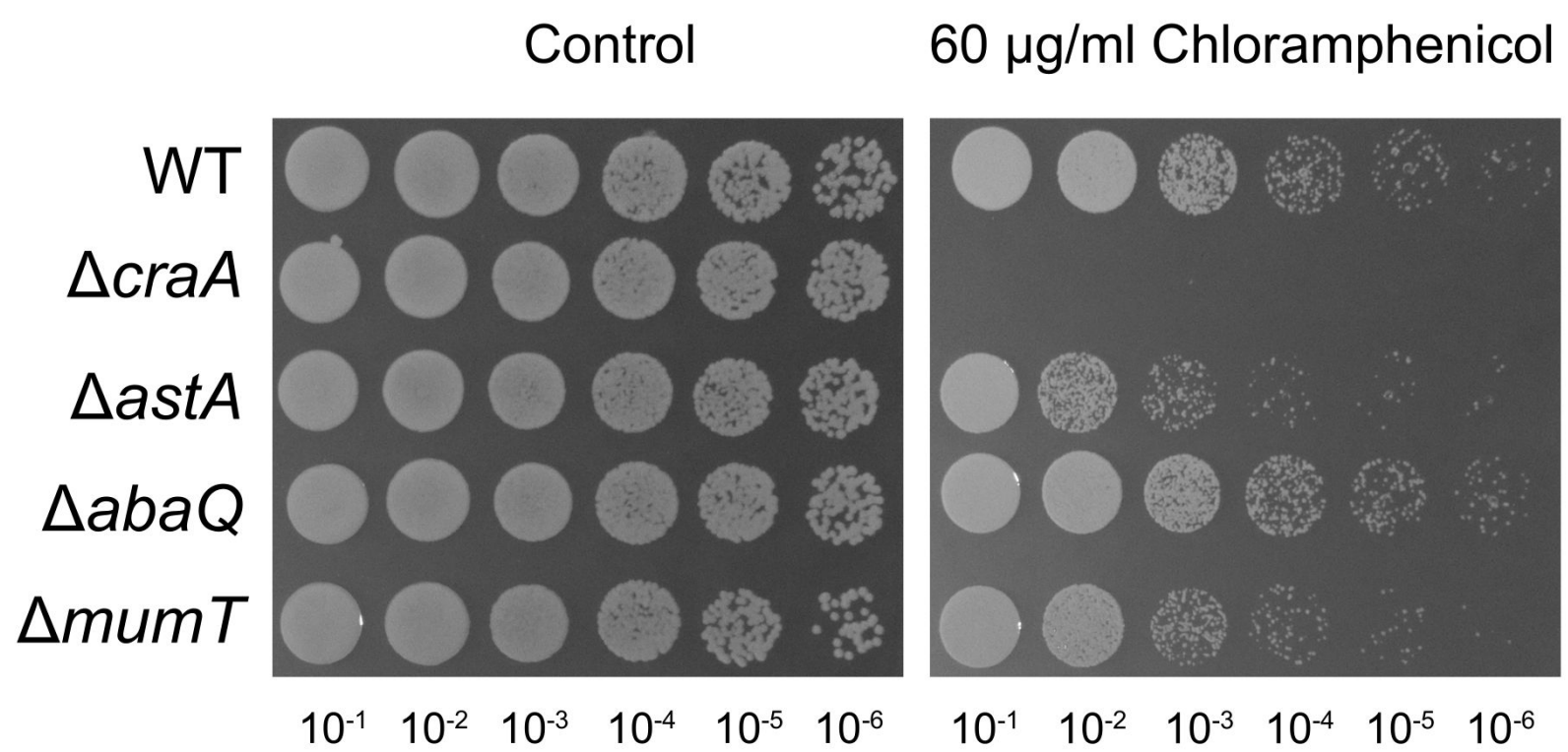

Supplementary Fig. 3. Growth kinetics of *A. baumannii* WT and efflux pump knockout strains in LB medium supplemented with sodium nitroprusside (SNP). (A) Growth curves in LB containing 16 mM SNP. (B-D) Quantification of growth parameters in LB medium supplemented with 16 mM or 32 mM SNP: (B) lag time (h), (C) maximum growth rates (μ_{max}, h^{-1}), and (D) doubling time (T_d, h). Black dots represent individual biological replicates; bars denote mean ± SEM from at least three independent experiments. Statistical significance was assessed using general linear hypothesis testing with Tukey's multiple comparisons. ****P* < 0.001; ***P* < 0.01; **P* < 0.05.

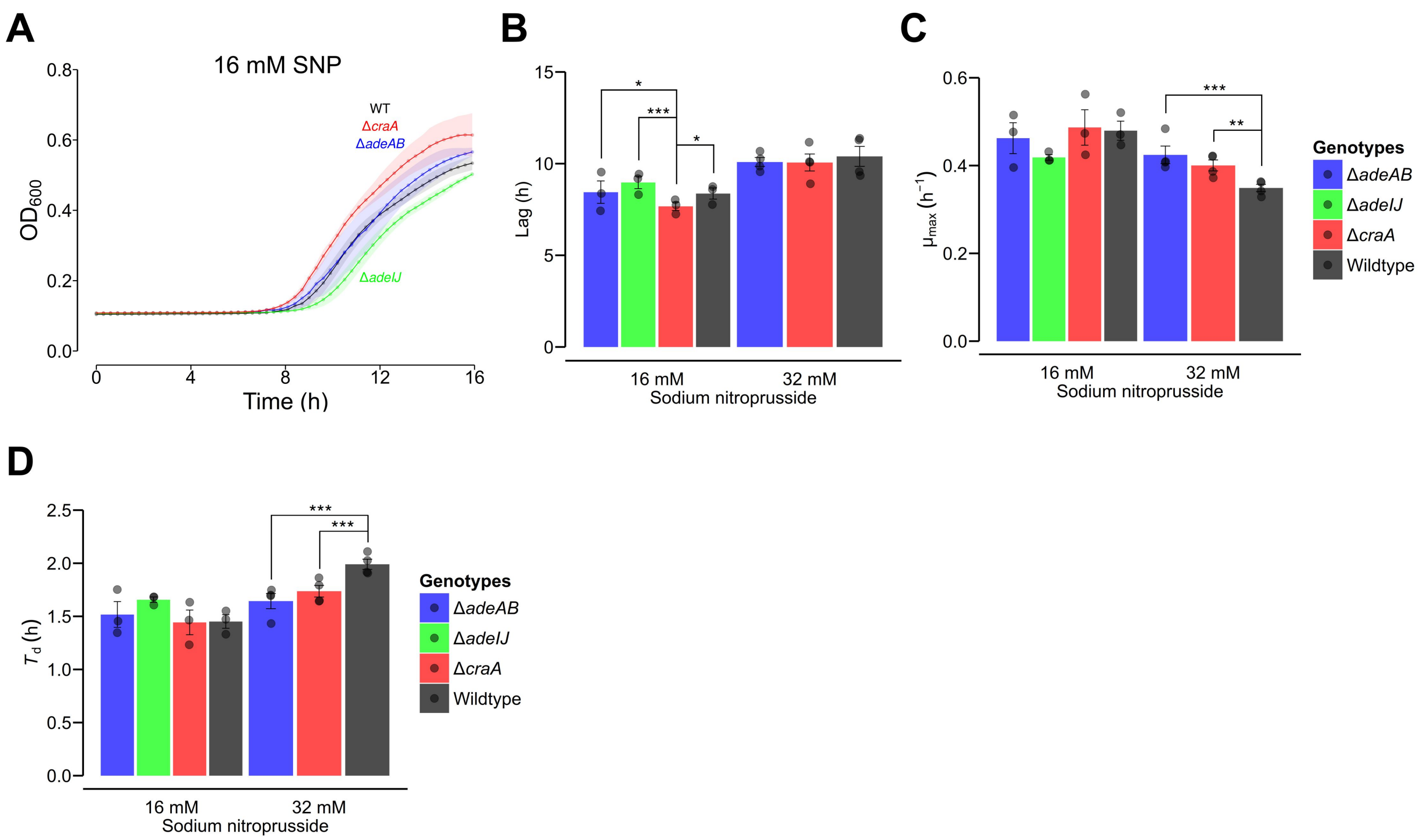

Supplementary Fig. 4. Growth parameters of *A. baumannii* WT and knockout strains cultured in LB medium supplemented with 0.5 mM H_2O_2 or 1.25 mM $CuSO_4$. (A) Maximum growth rates (μ_{max}, h^{-1}); (B) lag time (h); (C) Time to reach μ_{max} ($T_{\mu max}$, h); (D) Maximum optical density at 16 h (OD_{max}); (E) Area under the growth curve (AUC). Data represent individual biological replicates (black dots) and the mean ± SEM from at least three independent biological experiments. Statistical significance was determined using general linear hypothesis testing followed by Tukey's multiple comparison test. ***$P < 0.001$; **$P < 0.01$; *$P < 0.05$.

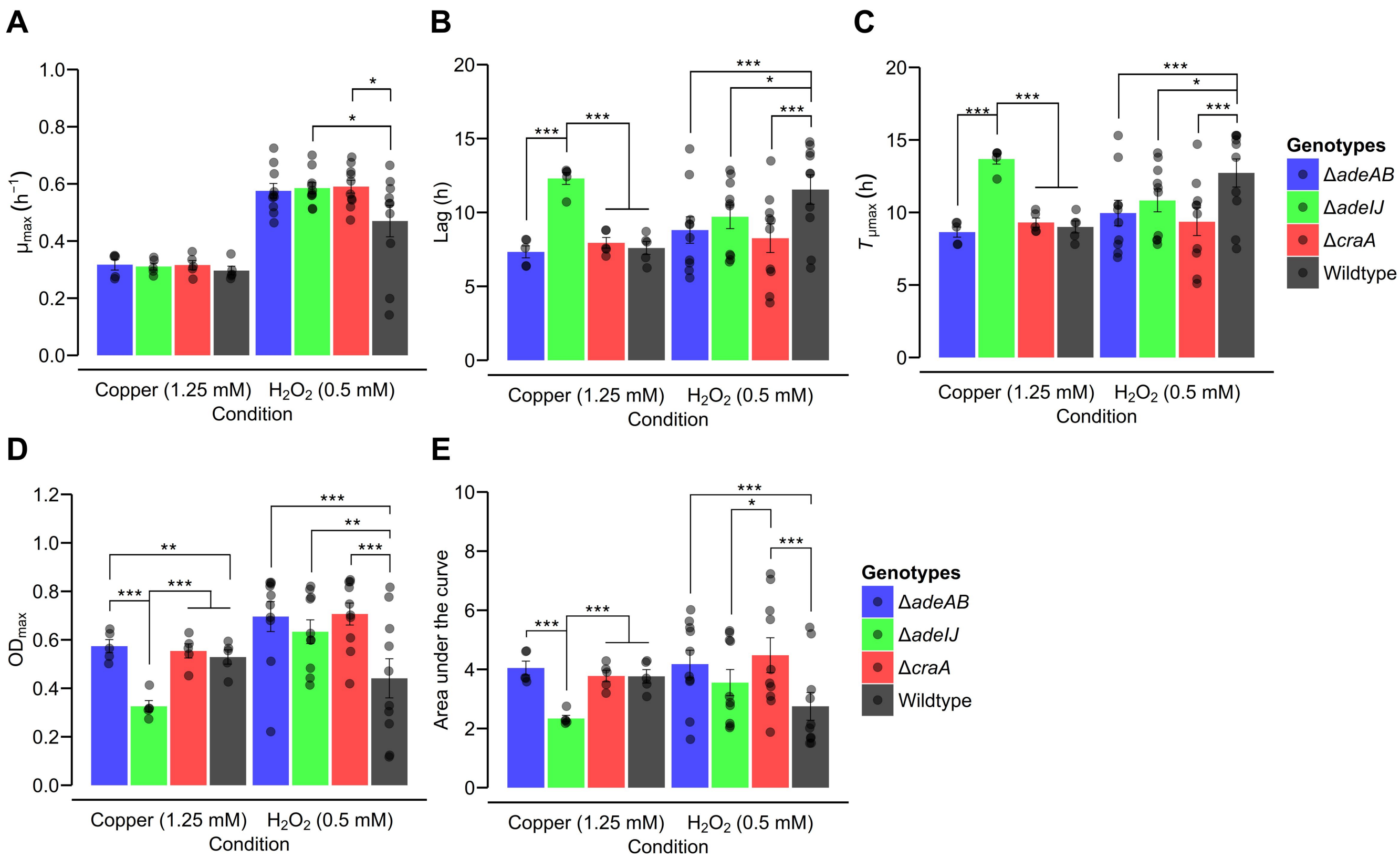

Supplementary Fig. 5. Representative inhibition zones on LB agar plates containing 2.5% H_2O_2, using bacterial cultures inoculated at different cell densities and incubation temperatures: (A) OD_{600} = 0.6, at 25°C; (B) OD_{600} = 0.06, at 25°C; (C) OD_{600} = 0.6, at 37°C; and (D) OD_{600} = 0.06, at 37°C. (E) Quantification of growth inhibition zones on LB agar plates containing H_2O_2 at 37°C. Statistical significance was determined using general linear hypothesis testing followed by Tukey's multiple comparison test (***, $P < 0.001$; **, $P < 0.01$; *, $P < 0.05$).

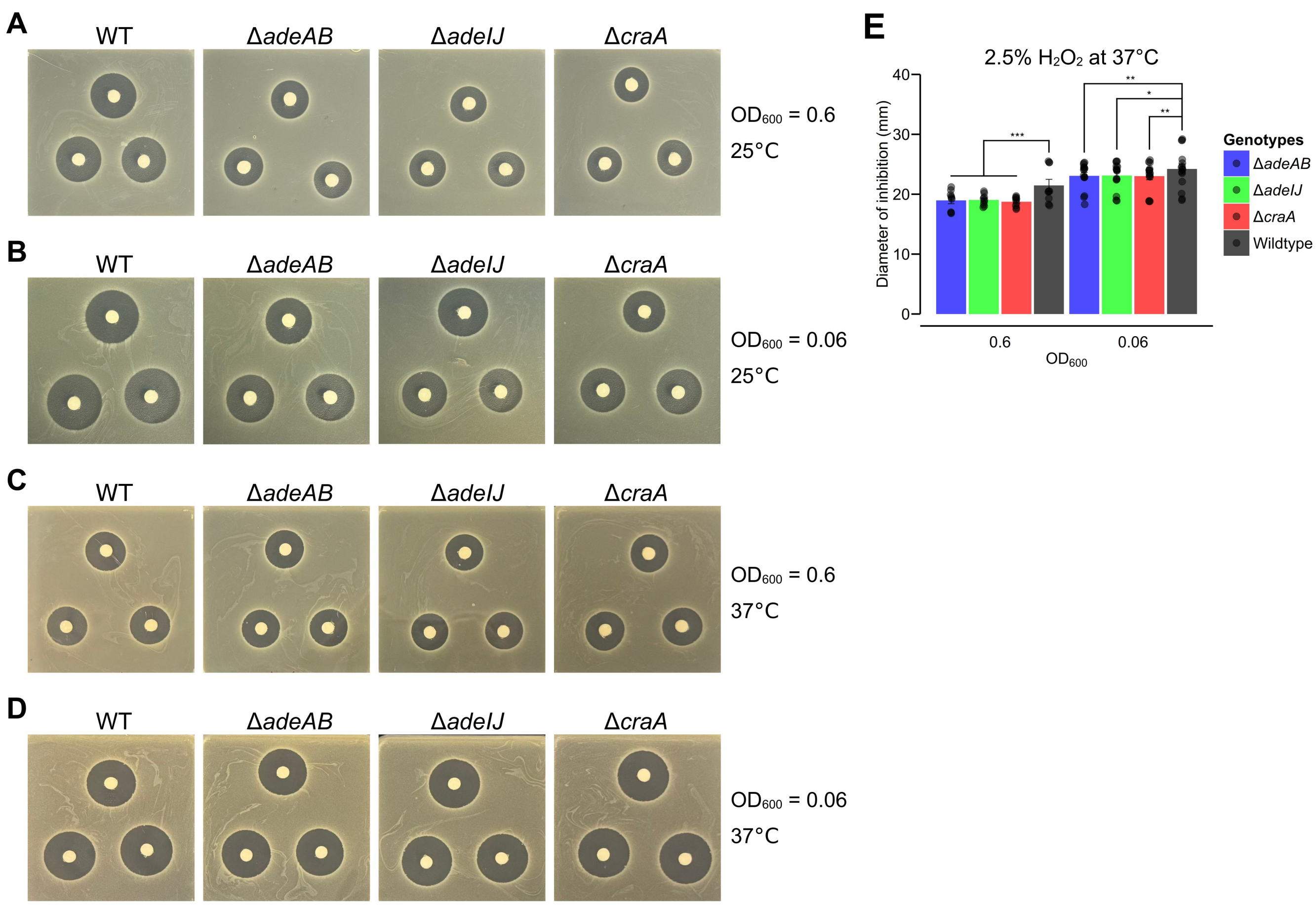

Supplementary Fig. 6. Growth kinetics of *A. baumannii* ATCC19606 wildtype and efflux pump knockout strains in LB medium supplemented with (A) 4 mM or (B) 8 mM manganese. Data represent means ± SEM from three independent biological replicates.

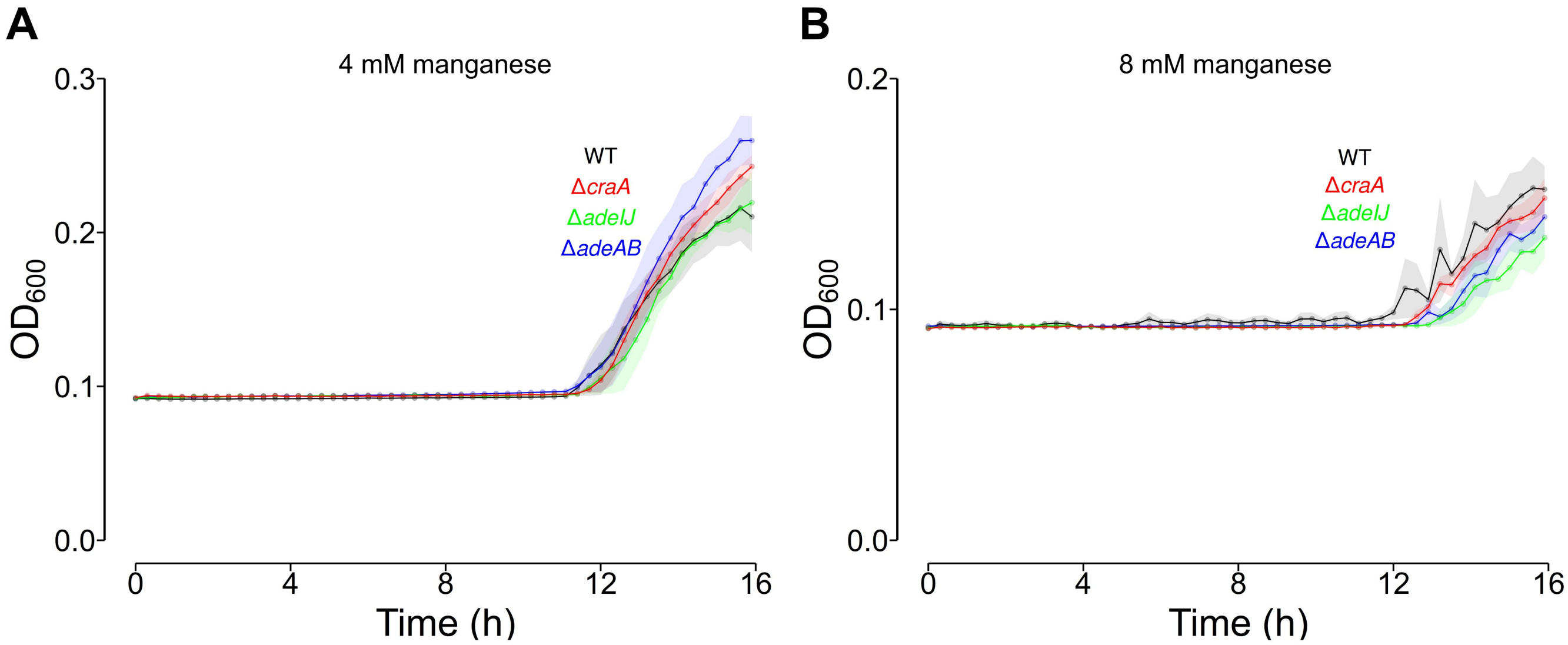

Supplement: Supplementary file 1 [file msystems.00009-26-s0002.pdf]
