# Supplementary material for: Chloramphenicol stress triggers oxidative adaptation in Acinetobacter baumannii ATCC19606 devoid of RND efflux pumps AdeAB or AdeIJ
Source: mSystems. 2026 Sep 3;11(9):e00009-26. doi: 10.1128/msystems.00009-26 (PMC13595967; doi:10.1128/msystems.00009-26)
Supplement: Supplementary file 2 [file reviewer-comments.pdf]

Chloramphenicol Stress Triggers Oxidative Adaptation in *Acinetobacter baumannii* ATCC19606 Devoid of RND efflux pumps AdeAB or AdeIJ

Wuen Ee Foong, Xinxin Xiang, Wenjun He, Xuan Yan, Jiabin Huang, Klaas Pos, and Heng-Keat Tam

Corresponding Author(s): Heng-Keat Tam, University of South China Hengyang Medical School

Review Timeline:

Submission Date:	January 7, 2026
Editorial Decision:	February 18, 2026
Revision Received:	May 14, 2026
Editorial Decision:	June 14, 2026
Revision Received:	June 22, 2026
Accepted:	July 15, 2026

Editor: Maria Scrascia

Reviewer(s): Disclosure of reviewer identity is with reference to reviewer comments included in decision letter(s). The following individuals involved in review of your submission have agreed to reveal their identity: Innocent Afeke (Reviewer #1)

Transaction Report:

(Note: With the exception of the correction of typographical or spelling errors that could be a source of ambiguity, letters and reports are not edited. The original formatting of letters and referee reports may not be reflected in this compilation.)

DOI: https://doi.org/10.1128/msystems.00009-26

Re: mSystems00009-26 (**Chloramphenicol Stress Triggers Oxidative Adaptation in *Acinetobacter baumannii* Devoid of Efflux Pumps**)

Dear Dr. Heng-Keat Tam:

Thank you for the privilege of reviewing your work. Below you will find my comments, instructions from the mSystems editorial office, and the reviewer comments.

The manuscript focuses on the role of 3 efflux pumps in antibiotic resistance of Acinetobacter baumannii under selective pressure of chloramphenicol. The topic is undoubtedly of general scientific interest, however a rigorous interpretation and contextualisation of results and the description of experimental plans and methods should be more precisely written. Based on major and minor issues highlighted by the reviewers, I strongly invite the authors to improve the manuscript taking into account each reviewers' concern.

Please return the manuscript within 60 days; if you cannot complete the modification within this time period, please contact me. If you do not wish to modify the manuscript and prefer to submit it to another journal, notify me immediately so that the manuscript may be formally withdrawn from consideration by mSystems.

Revision Guidelines
To submit your modified manuscript, log into the submission site at https://msystems.msubmit.net/cgi-bin/main.plex. Go to Author Tasks and click the appropriate manuscript title to begin. The information you entered when you first submitted the paper will be displayed; update this as necessary. Note the following requirements:

• Upload point-by-point responses to the issues raised by the reviewers in a file named "Response to Reviewers," NOT in your cover letter.
• Upload a compare copy of the manuscript (without figures) as a "Marked-Up Manuscript" file.
• Upload a clean .DOC/.DOCX version of the revised manuscript and remove the previous version.
• Each figure must be uploaded as a separate, editable, high-resolution file (TIFF or EPS preferred), and any multipanel figures must be assembled into one file.
• Any supplemental material intended for posting by ASM should be uploaded with their legends separate from the main manuscript. You can combine all supplemental material into one file (preferred) or split it into a maximum of 10 files with all associated legends included.

For complete guidelines on revision requirements, see our Submission and Review Process webpage. Submission of a paper that does not conform to guidelines may delay acceptance of your manuscript.

Data availability: ASM policy requires that data be available to the public upon online posting of the article, so please verify all links to sequence records, if present, and make sure that each number retrieves the full record of the data. If a new accession number is not linked or a link is broken, provide mSystems production staff with the correct URL for the record. If the accession numbers for new data are not publicly accessible before the expected online posting of the article, publication may be delayed; please contact production staff (mSystems@asmusa.org) immediately with the expected release date.

Publication Fees: For information on publication fees and which article types are subject to charges, visit our website. If your manuscript is accepted for publication and any fees apply, you will be contacted separately about payment during the production process; please follow the instructions in that e-mail. Arrangements for payment must be made before your article is published.

ASM Membership: Corresponding authors may join or renew ASM membership to obtain discounts on publication fees. Need to upgrade your membership level? Please contact Customer Service at Service@asmusa.org.

The ASM Journals program strives for constant improvement in our submission and publication process. Please tell us how we can improve your experience by taking this quick Author Survey.

Thank you for submitting your paper to mSystems.

Sincerely,
Maria Scrascia
Editor
mSystems

Reviewer #1 (Comments for the Author):

I have the following major and minor concerns.

Major Concerns
1.Misleading Title
I am of the view that the title is misleading. The phrase "Devoid of Efflux Pumps" implies the complete absence of efflux systems, whereas the study investigates strains with specific efflux pump deletions (e.g., AdeAB). The title should be revised to accurately reflect the scope of the experimental work.
2.Methodological Clarity and Validation
Key methodological details require clarification, particularly regarding the construction, confirmation, and stability of the efflux pump deletion mutants. In addition, the use of appropriate controls to validate that the observed phenotypes are directly attributable to efflux pump loss is insufficiently described.
3.Overinterpretation of Findings
The conclusions drawn in several sections appear broader than what the data can robustly support. Claims relating to resistance mechanisms should be moderated and strictly aligned with the experimental evidence presented.
4.Limited Contextualisation of Results
The discussion does not sufficiently engage with existing literature, particularly with respect to compensatory resistance mechanisms and the limitations inherent in analysing single or selected efflux pump deletions. This weakens the broader relevance of the findings.

Minor Concerns
1.Rationale for Efflux Pumps and Antibiotic Selection
Could the authors clarify the rationale for selecting the specific efflux pumps and antibiotics investigated? A clearer justification would improve the coherence and scientific motivation of the study.
2.Terminology and Precision of Language
Some terms and phrases overstate the biological implications of the findings. Greater precision in language would improve clarity and prevent potential misinterpretation by readers.
3.Presentation and Flow of the Discussion
The discussion could be streamlined to improve logical flow, avoid repetition, and better distinguish between results directly supported by data and speculative interpretations.

Reviewer #2 (Comments for the Author):

This was a very nicely written manuscript that describes the role of different efflux pumps in Acinetobacter baumannii, with a focus on chloramphenicol resistance. Chloramphenicol is not a drug that is prescribed for treating A. baumannii, and the organism is intrinsically resistant to this drug. However, the interplay of efflux pumps and their effect on the organism is an interesting topic and the authors have performed their work really nicely.
I have only minor points for the authors to consider.

1) Line 76. Global, not goal
2) MIC determination. MICs are usually performed using standard methods from EUCAST or CLSI, whereby isolates are tested in Mueller Hinton media. Using LB is not a standard MIC test. Also the inoculum is on the high side, and I wonder if there is an inoculation effect which leads to a higher MIC value. Why was this method chosen and not the standard methods?
3) Line 199. The susceptibility testing and growth curves were performed at 37C; why were the isolates grown at 25C in the H2O2 assay? Would there be a difference if grown at 37C?
4) When you knocked out the adeAB pump, what happened to their regulators adeRS? Similarly, when the other pumps were knocked out, was there any effect on adeRS? On a similar note, what about adeN?
5) Line 438. I do not think that the conditions are bacteriostatic. You are using a subinhibitory concentration of chloramphenicol, and the isolates are able to replicate (if they were unable to replicate, you would be at an inhibitory concentration)
6) Line 445. adeAB are on an operon. Was the adeA increase the same as the adeB increase? The same applies for adeIJ.
7) Is there anything known about the regulator of craA?
8) Line 484. The sentence beginning "Despite inactivation of abuO and ompA.." I find it hard to follow. Can you rephrase this please.
9) I would be interested in hearing how the knockout strains accumulate substrate like ethidium bromide compared to wt. Presumably this is older data, but it could still be discussed.
10) Lines 651-654: are there any data on increased expression of T6SS and reduced antimicrobial susceptibility?
11) Table 1. Can you explain the relevance of the bold font
12) Line 921. "E" is listed twice. I think the second time it should be an "F"
13) I am struggling to understand figure 2. Can the data be summarised in a less complicated way?
14) Figure 7: I really like the concept of the schematic however it is misleading as it suggests that craA acts from the cytosol and crosses over the outer membrane

We have addressed the comments from the reviewers and the answers given are in blue letter-color (please refer also to the tracked changes documents):

Reviewer #1 (Comments for the Author):

I have the following major and minor concerns.

Major Concerns

1.Misleading Title
I am of the view that the title is misleading. The phrase "Devoid of Efflux Pumps" implies the complete absence of efflux systems, whereas the study investigates strains with specific efflux pump deletions (e.g., AdeAB). The title should be revised to accurately reflect the scope of the experimental work.

We agree that the title might have been too generic, we have now revised the title to accurately reflect the scope of the work.

“Chloramphenicol Stress Triggers Oxidative Adaptation in *Acinetobacter baumannii* Devoid of RND Efflux Pumps AdeAB or AdeIJ”.

2.Methodological Clarity and Validation
Key methodological details require clarification, particularly regarding the construction, confirmation, and stability of the efflux pump deletion mutants. In addition, the use of appropriate controls to validate that the observed phenotypes are directly attributable to efflux pump loss is insufficiently described.

Thank you for highlighting the need for clearer methodological description and validation of the deletion mutants. We have revised the Methods section to explicitly detail mutant construction, confirmation, and controls.

Briefly, efflux pump deletion mutants were generated by allelic exchange using suicide plasmids carrying upstream and downstream homologous regions, followed by antibiotic selection and sucrose counter-selection. Correct gene replacement was first verified by PCR and Sanger sequencing across the recombination junctions. To further ensure genetic integrity and exclude secondary mutations, all strains including wildtype, Δ*adeAB*, Δ*adeIJ*, and Δ*craA*, were subsequently validated by whole-genome sequencing. Furthermore, the stability of the mutant strains have been verified before and after the transcriptomic assays. This information has been incorporated as following:

Lines 156-175 (marked-up version):

“Briefly, targeted gene deletion mutants (e.g. *abaQ*, *adeAB*, *adeIJ*, *astA*, *craA*, and *mumT*) were generated by allelic exchange using suicide plasmids carrying upstream and downstream homologous regions, followed by antibiotic selection and sucrose counter-selection as previously described [24. Foong 2019; 25. Amin 2013]. PCR products of upstream and downstream homologous region of the *adeAB*, *adeIJ* or *craA* genes were cloned into pBIISK_sacB/kanR by Gibson assembly……… Upstream and downstream homologous region of the *abaQ*, *astA*, and *mumT* genes were cloned into pMo130-Tel^R by

Gibson assembly…….. Counter-selection was performed on LB + 15% sucrose. Correct gene replacement was first verified by PCR and Sanger sequencing across the recombination junctions. All primers used are listed in Supplementary Table S1."

Lines 177-199 (marked-up version):

"Whole genome sequencing

Whole-genome sequencing was performed to confirm the genetic integrity of *A. baumannii* ATCC19606 wildtype and the isogenic deletion mutants Δ*adeAB,* Δ*adeIJ*, and Δ*craA*. Genomic DNA from each strain was fragmented to an average size of ~350 bp using a Covaris LE220R-plus system (Covaris, USA), followed by end repair, A-tailing, adapter ligation, and PCR amplification. Libraries were sequenced on Illumina platforms (150 bp paired-end) by Novogene Bioinformatics Technology Co., Ltd. (Beijing, China).

Reads were quality-trimmed using Trimmomatic v0.39 [30. Bolger 2014] and assembled *de novo* with SPAdes v3.15.5 [31. Bankevich 2012]. The assemblies comprised 22, 40, 38, and 35 contigs (> 500 bp) for WT, Δ*adeAB*, Δ*adeIJ*, and Δ*craA*, respectively. Contigs with significant similarity to plasmid sequences, identified by BLASTn searches against the NCBI nucleotide database were excluded from chromosomal reconstruction [32. Altschul 1990, 33. Sayers 2025]. The remaining contigs were scaffolded using RagTag [34. Alonge 2022]. Scaffolded chromosome assemblies and putative extrachromosomal contigs have been deposited in DDBJ/ENA/GenBank under accession numbers JBWVBS000000000–JBWVBV000000000.".

For variant analysis, reads from each mutant were mapped to the wildtype assembly and analyzed independently using Snippy v4.6.0 (https://github.com/tseemann/snippy) and freebayes v1.3.6 [35. Garrison 2012], retaining only SNPs and InDels identified by both pipelines. Structural variants were detected through pairwise assembly comparison using MUMmer [36. Marçais 2018] and Assemblytics [37. Nattestad 2016]."

To ensure that the observed phenotypes were attributable to efflux pump loss rather than general growth defects, the wildtype strain was used as the reference control in all phenotypic assays. Growth on drug-free LB medium verified baseline viability, whereas drug-containing medium assessed resistance phenotypes under identical conditions. We incorporated the following statement "*A. baumannii* wildtype (control) and efflux pump knockout strains" and "Drug-free LB medium served as the control." in the method to clarify the use of appropriate experimental controls.

Additionally, we included a structural variant analysis of targeted gene deletions in the results, as follows:

Lines 374-388 (marked-up version):

"Structural variant analysis confirms targeted gene deletions

Structural variant analysis confirmed a single, targeted deletion in each engineered strain relative to wildtype. In Δ*adeAB*, a 4,282-bp deletion (positions 1,844,323–1,848,605; CP059040) removes nearly the entire *adeAB* operon, encompassing *adeA* (H0N29_08675; 1,844,319–1,845,509) and *adeB* (H0N29_08680; 1,845,506–1,848,616). The deletion begins

4 bp within *adeA* and terminates 11 bp upstream of the *adeB* stop codon. In Δ*adeIJ*, a 4,443-bp deletion (positions 737,224–741,667; CP059040) removes the reverse-strand *adeIJ* operon, including *adeJ* (H0N29_03545; complement 737,233–740,409) and *adeI* (H0N29_03550; complement 740,422–741,672). The breakpoints are located 9 bp upstream of *adeJ* and 5 bp before the end of *adeI*. In Δ*craA*, a 1,157-bp deletion (positions 364,652–365,809; CP059040) removes the central region of the *craA* coding sequence (H0N29_01695; 364,609–365,838), spanning from 43 bp downstream of the start codon to 29 bp upstream of the stop codon. Consensus variant analysis using Snippy and freebayes detected no additional SNPs or small InDels outside the intended deletions, confirming that each mutant differs from wildtype only at the targeted locus."

3.Overinterpretation of Findings

The conclusions drawn in several sections appear broader than what the data can robustly support. Claims relating to resistance mechanisms should be moderated and strictly aligned with the experimental evidence presented.

Thank you for this comment. We have toned down the discussion and conclusion in the revised manuscript.

For example,

Lines 746-752 (marked-up version):

"In contrast, Δ*adeAB* showed pronounced transcriptional remodeling, marked by upregulation of ribosomal proteins, transporters including CraA, and [Fe-S] cluster biogenesis (ISC system, *nfuA*, *grxD*), together with repression of propanoate metabolism (Fig. 4B; Fig. 5A-5C, Supplementary Table S2-S5). The mutant also exhibited increased tolerance to H_2O_2, suggesting that induction of stress-related pathways following *adeAB* deletion may confer cross-protection against oxidative stress."

Conclusion, Lines 863-867 (marked-up version):

"Our findings provide a comprehensive view of strain-specific adaptations of ATCC19606 to chloramphenicol stress, establishing CraA as the principal chloramphenicol efflux determinant, while indicating potential links between RND inactivation and oxidative as well as metal stress responses (Fig. 7).

4.Limited Contextualisation of Results

The discussion does not sufficiently engage with existing literature, particularly with respect to compensatory resistance mechanisms and the limitations inherent in analysing single or selected efflux pump deletions. This weakens the broader relevance of the findings.

Thank you for this constructive comment. We agree that placing our findings more explicitly within the context of previously described compensatory resistance mechanisms strengthens the broader relevance of the study.

Compensatory and redundant efflux responses have been documented in several bacterial species. In *Escherichia coli*, disruption of the AcrAB–TolC system can result in upregulation of

alternative efflux pumps, illustrating the plasticity of multidrug resistance networks ((Alon Cudkowicz 2019, Shuster 2016). Similarly, in *A. baumannii* ATCC17978, inactivation of *adeFGH* was reported to increase *adeAB* expression (Leus 2018), supporting the concept of cross-regulation among RND systems. In addition, transcriptomic analyses in AB5075 have shown that efflux perturbation can be accompanied by broader metabolic and regulatory adjustments (Leus 2020).

Our observation that *craA* is upregulated in the Δ*adeAB* background, even in the absence of chloramphenicol (Fig. 5B; Dataset 1), is consistent with these reports and supports the idea of a coordinated efflux network with partially overlapping substrate specificities. At the same time, we acknowledge that analysis of single-gene deletions has inherent limitations, as functional redundancy may mask phenotypic consequences and regulatory interactions may differ between laboratory and clinical strains. The relatively modest transcriptomic perturbations observed in some mutants in our study are in line with this possibility.

To address this point, we have revised the discussion to more explicitly reference these studies and to frame our findings within the broader context of compensatory resistance mechanisms. We have also added the following statement to the conclusion:

We have revised the text as following:

Discussion, Lines 807-818 (marked-up version):

"Notably, *craA* was also upregulated in the Δ*adeAB* background even in the absence of chloramphenicol (Fig. 5B; Dataset 1). This observation aligns with findings in strain ATCC17978, where inactivation of *adeFGH* led to overexpression of *adeAB* [70. Leus 2018], and with the broader concept of a coordinated, versatile bacterial efflux network comprising RND and MFS with overlapping specificities [71. Alon Cudkowicz 2019, 72. Shuster 2016]. Collectively, the data support a hierarchical efflux model in which CraA plays a primary role in chloramphenicol efflux, whereas AdeIJK may contribute to redox and metal homeostasis (Fig. 7), with potential compensatory or synergistic interactions between RND systems and CraA under antibiotic stress."

Conclusion, Lines 863-872 (marked-up version):

"Our findings provide a comprehensive view of strain-specific adaptations of ATCC19606 to chloramphenicol stress, establishing CraA as the principal chloramphenicol efflux determinant, while indicating potential links between RND inactivation and oxidative as well as metal stress responses (Fig. 7). The observed connections among efflux regulation, central metabolism, and stress physiology point to the complexity of intrinsic antibiotic resistance and underscore the importance of accounting for strain-specific variation in *A. baumannii*. Future work incorporating combinatorial deletions, regulatory mutants, and clinically derived isolates would help clarify the hierarchy and interplay of efflux systems under antibiotic stress."

We believe these revisions provide clearer contextualisation and appropriately moderate the generalizability of our conclusions.

References:

1. Leus, 2018. Substrate specificities and efflux efficiencies of RND efflux pumps of *Acinetobacter baumannii*. *J. Bacteriol*. 200, e00049-18.
2. Alon Cudkowicz, 2019. Deletion of the major *Escherichia coli* multidrug transporter AcrB reveals transporter plasticity and redundancy in bacterial cells. PLoS One 14, e0218828.
3. Shuster, 2016. A Transporter interactome is essential for the acquisition of antimicrobial resistance to antibiotics. PLoS One 11(4):e0152917.
4. Leus, 2020. Inactivation of AdeABC and AdeIJK efflux pumps elicits specific nonoverlapping transcriptional and phenotypic responses in *Acinetobacter baumannii*. Mol Microbiol 114:1049–1065.

Minor Concerns

1.Rationale for Efflux Pumps and Antibiotic Selection

Could the authors clarify the rationale for selecting the specific efflux pumps and antibiotics investigated? A clearer justification would improve the coherence and scientific motivation of the study.

Thank you for this helpful suggestion. We have clarified the rationale for selecting the efflux pumps and antibiotic in the introduction.

We focused on the RND transporters AdeABC and AdeIJK and the MFS transporter CraA because they represent the principal chloramphenicol efflux determinants in *Acinetobacter baumannii* and differ markedly in conservation and physiological roles (refer to Magnet 2001; Damier-Piolle 2008; Foong 2025). AdeIJK is highly conserved across the *Acinetobacter* genus and is considered part of the intrinsic resistome (Darby 2023; Naidu 2023), suggesting a core physiological function. In contrast, AdeABC is enriched in clinical isolates and associated with acquired multidrug resistance (Darby 2023; Naidu 2023), whereas CraA is broadly conserved in *A. baumannii* and is the major chloramphenicol exporter (Lin 2017).

Although chloramphenicol is not commonly used to treat *A. baumannii* infections due to intrinsic resistance, it is one of the best-characterized substrates of these pumps and provides a well-defined model to dissect efflux-mediated physiology. Because AdeABC, AdeIJK, and CraA share overlapping substrate profiles but distinct evolutionary distributions, we used chloramphenicol as a controlled probe to determine how loss of each system reshapes resistance and stress adaptation.

We have revised the introduction accordingly:

Lines 115-143 (marked-up version):

"To examine adaptive antibiotic response to chloramphenicol, we performed RNA-seq on wildtype *A. baumannii* ATCC19606 and mutants lacking the Resistance Nodulation cell Division-type (RND) efflux pumps AdeAB or AdeIJ. Chloramphenicol was selected as a model antibiotic because prior studies identified AdeABC, AdeIJK and the Major Facilitator Superfamily (MFS) efflux pump CraA as major resistance determinants in this strain [19. Magnet 2001; 20. Damier-Piolle 2008; 21. Foong 2025]. While *adeIJK* is conserved across the *Acinetobacter* genus, suggesting a broader physiological function, *adeABC* is variably distributed [22. Darby 2023; 23. Naidu 2023], pointing to distinct evolutionary trajectories.

Supporting this functional divergence, previous work in strains AB5075 and AYE showed that *adeB* inactivation alters motility, competence, type IV pilus genes, iron acquisition, and biofilm-associated pathways, including poly-β-1,6-N-acetyl-D-glucosamine (*pga*) biosynthesis. Similarly, disruption of *adeIJK* has been linked to changes in motility, *pga*-dependent biofilm formation, lipid synthesis, efflux networks, and fatty acid, iron, and carbon metabolism genes [Leus 2020]. Given their overlapping substrate profiles but divergent evolutionary histories, we hypothesized that deletion of these efflux pumps would trigger strain-specific resistance adaptations under chloramphenicol stress. Consistent with this broader physiological role of RND pumps, our data show that *craA* is expressed at low basal levels in wildtype ATCC19606 strain, whereas Δ*adeAB* and Δ*adeIJ* mutants display distinct regulatory and metabolic remodeling under chloramphenicol stress. Together, these findings reinforce the view that efflux systems in *A. baumannii* function not only as drug exporters but also as nodes within interconnected metabolic and stress-response networks that shape adaptive resistance."

References:

1. Magnet, 2001. Resistance-nodulation-cell division-type efflux pump involved in aminoglycoside resistance in *Acinetobacter baumannii* strain BM4454. Antimicrob Agents Chemother.2001;45:3375–80.

2. Damier-Piolle, 2008. AdeIJK, a resistance-nodulation-cell division pump effluxing multiple antibiotics in *Acinetobacter baumannii*. Antimicrob Agents Chemother 2008;52:557–62.

3. Foong, 2025. Molecular determinants of substrate specificity in the efflux pump CraA from *Acinetobacter baumannii*. Microbiol Spectr 13:e0111925.

4. Darby, 2023. RND pumps across the genus *Acinetobacter*: AdeIJK is the universal efflux pump. Microb Genom. 9, mgen000964.

5. Naidu, 2023. Evolution of RND efflux pumps in the development of a successful pathogen. Drug Resist Updat. 66, 100911.

6. Lin, 2017. Distribution of different efflux pump genes in clinical isolates of multidrug-resistant *Acinetobacter baumannii* and their correlation with antimicrobial resistance. J Microbiol Immunol. Infect. 50, 224–231.

7. Leus, 2020. Inactivation of AdeABC and AdeIJK efflux pumps elicits specific nonoverlapping transcriptional and phenotypic responses in *Acinetobacter baumannii*. Mol Microbiol 114:1049–1065.

2.Terminology and Precision of Language

Some terms and phrases overstate the biological implications of the findings. Greater precision in language would improve clarity and prevent potential misinterpretation by readers.

We appreciate the reviewer's comment regarding terminology and the need for greater precision in describing the biological implications of our findings. In response, we have carefully revised the manuscript to moderate statements that could be interpreted as overstating causality or generalizability.

Specifically, we have replaced definitive language with more cautious phrasing (e.g., "may", "potential", and "suggest") where appropriate. In several instances, conclusions have been reframed to emphasize that our data support particular interpretations but do not conclusively

demonstrate direct mechanistic links. Where relevant, we now explicitly acknowledge alternative explanations and clarify the distinction between data-driven observations and speculative interpretation.

These revisions aim to improve clarity, avoid overextension of the results, and more accurately reflect the scope and limitations of our experimental design, particularly given that our conclusions are based on single-gene deletions and transcriptomic analyses under defined laboratory conditions.

We believe these changes enhance the precision and balance of the manuscript while preserving the central findings.

Lines 501-506 (marked-up version):

Together, these findings point to functional differences among RND efflux pumps and **suggest** that, while AdeIJK **may** play a broad-spectrum detoxification and membrane remodeling, AdeAB could have roles extending beyond drug efflux, potentially influencing metabolic and regulatory homeostasis.

Lines 574-578 (marked-up version):

Furthermore, induction of *adeA* and *adeB* under chloramphenicol exposure appeared to coincide with increased *craA* expression (Fig. 5G; Supplementary Dataset 1), **suggesting** a coordinated or potentially complementary efflux activity [28. Foong 2019].

Lines 750-752 (marked-up version):

The Δ*adeAB* mutant also exhibited increased tolerance to H_2O_2, **suggesting** that induction of stress-related pathways following *adeAB* deletion may confer cross-protection against oxidative stress.

Conclusion, Lines 863-867 (marked-up version):

Our findings provide a comprehensive view of strain-specific adaptations of ATCC19606 to chloramphenicol stress, establishing CraA as the principal chloramphenicol efflux determinant, while indicating **potential** links between RND inactivation and oxidative as well as metal stress responses (Fig. 7).

3.Presentation and Flow of the Discussion
The discussion could be streamlined to improve logical flow, avoid repetition, and better distinguish between results directly supported by data and speculative interpretations.

We thank the reviewer for this helpful suggestion. We have carefully revised the Discussion to improve clarity, coherence, and readability. Specifically, we:

1. Streamlined the narrative structure by reorganizing the section into a clearer progression:
(i) stress-response functions of RND systems,
(ii) chloramphenicol resistance hierarchy, and

(iii) physiological implications beyond drug efflux

2. Removed repetitive statements describing efflux-mediated resistance and consolidated overlapping paragraphs.

3. Clearly separated evidence-based conclusions from interpretation, ensuring that transcriptomic and phenotypic observations are presented first, followed by explicitly identified hypotheses or broader implications.

4. Reduced speculative language and limited interpretations to those directly supported by experimental data.

These revisions improve logical flow and better align the Discussion with the results presented in the manuscript.

Reviewer #2 (Comments for the Author):

This was a very nicely written manuscript that describes the role of different efflux pumps in Acinetobacter baumannii, with a focus on chloramphenicol resistance. Chloramphenicol is not a drug that is prescribed for treating A. baumannii, and the organism is intrinsically resistant to this drug. However, the interplay of efflux pumps and their effect on the organism is an interesting topic and the authors have performed their work really nicely.

We like to thank the reviewer for the in-depth review of the manuscript.

I have only minor points for the authors to consider.
1) Line 76. Global, not goal

We apologize for this error and have corrected it accordingly.

2) MIC determination. MICs are usually performed using standard methods from EUCAST or CLSI, whereby isolates are tested in Mueller Hinton media. Using LB is not a standard MIC test. Also the inoculum is on the high side, and I wonder if there is an inoculation effect which leads to a higher MIC value.

Thank you for this important comment. We do not believe the elevated MIC values result from an inoculum effect. In inoculum-dependent artefacts, growth typically accelerates and reaches stationary phase earlier. In our growth-curve analyses (Fig. 1A), neither lag phase nor time to stationary phase shifted in a manner consistent with inoculum-driven resistance, indicating that the observed MIC differences reflect genuine physiological effects rather than cell-density bias.

Why was this method chosen and not the standard methods?

We agree that EUCAST and CLSI protocols using Mueller–Hinton medium represent the reference standard for clinical susceptibility testing. However, the purpose of our study was not to determine clinical breakpoints but to compare relative susceptibility among isogenic laboratory mutants under controlled experimental conditions.

LB medium was selected for two reasons:

1. Experimental consistency – all phenotypic, transcriptomic, and stress-response assays in this study were performed in the same medium to avoid medium-dependent physiological variation.
2. Comparability with prior mechanistic work – our previous investigations of wildtype and efflux pump mutants, as well as studies from other groups, were conducted under identical conditions, allowing for direct comparison with established datasets (Leus 2018; Foong 2019; Foong 2025).

Accordingly, MIC values in this study should be interpreted as relative phenotypic measurements within a defined experimental framework, rather than clinical susceptibility classifications.

References:

1. Foong, 2019. The chloramphenicol/H^+ antiporter CraA of *Acinetobacter baumannii* AYE reveals a broad substrate specificity. *J. Antimicrob. Chemother.* 74, 1192–1201.

2. Foong, 2025. Molecular determinants of substrate specificity in the efflux pump CraA from *Acinetobacter baumannii*. *Microbiol. Spectr.* 13, e0111925.

3. Leus, 2018. Substrate specificities and efflux efficiencies of RND efflux pumps of *Acinetobacter baumannii*. *J. Bacteriol*. 200, e00049-18.

3) Line 199. The susceptibility testing and growth curves were performed at 37C; why were the isolates grown at 25C in the H2O2 assay? Would there be a difference if grown at 37C?

We thank the reviewer for this helpful question. In our initial experiments, the H_2O_2 susceptibility assay was performed at 37°C. Under these conditions, when cultures were inoculated at OD_{600} = 0.06, all strains reached nearly identical cell densities after 16–18 h of incubation, making it difficult to clearly distinguish the differences between the wildtype and efflux pump mutants. After performing a careful statistical analysis using a linear mixed-effects model with maximum likelihood estimation, incorporating a random intercept for each biological replicate, we did observe statistically significant differences at this inoculum. However, clearer differences in oxidative stress responses were observed when a higher inoculum (OD_{600} = 0.6) was used. As shown in Supplementary Fig. 4, the growth kinetics of the wildtype and knockout strains are not identical, which likely influenced the extent to which oxidative stress phenotypes could be resolved at higher temperatures.

To improve the ability to discriminate strain-specific differences in H_2O_2 susceptibility, we performed the disk diffusion assays at 25°C. At this temperature, bacterial growth remained robust, but it progressed more slowly, allowing for clearer separation of phenotypes between the wildtype and efflux pump mutants. Importantly, the overall trends in oxidative stress susceptibility were consistent between 25°C and 37°C, although the magnitude of the differences was more pronounced at 25°C. This temperature allowed us to better capture and distinguish strain-specific variations in susceptibility, providing a clearer assessment of the impact of efflux pump deletion on oxidative stress response.

To address the reviewer's question, the results of the H_2O_2 susceptibility assays performed at 37°C have now been incorporated into the revised manuscript (Supplementary Fig. 5).

Revised text added to the manuscript, Lines 692-694 (marked-up version):

"In agreement with these results, the wildtype exhibited greater growth inhibition on H_2O_2-containing solid medium at both 25°C and 37°C (Fig. 6C, Supplementary Fig. 5)"

4) When you knocked out the adeAB pump, what happened to their regulators adeRS? Similarly, when the other pumps were knocked out, was there any effect on adeRS? On a similar note, what about adeN?

Thank you for this valuable suggestion. We examined the transcription of the known efflux regulators *adeR*, *adeS*, and *adeN* in all knockout backgrounds and under chloramphenicol exposure. As shown in Dataset 1 and Fig. 5H, expression of *adeR*, *adeS*, and *adeN* remained largely unchanged in the Δ*adeAB*, Δ*adeIJ*, and Δ*craA* mutants compared with the wildtype, both in the presence and absence of chloramphenicol. Thus, deletion of individual pumps did not trigger compensatory transcriptional activation of their canonical regulatory systems.

This observation suggests that the adaptive response observed in our study does not rely on activation of classical efflux regulatory pathways but instead involves selective remodeling of alternative regulatory networks.

We have included the RNA-seq data (*adeS*, *adeR*, and *adeN*) in Fig. 5H.

Revised text added to the manuscript, Lines 629-641 (marked-up version):

"Given the established roles of transcriptional regulators such as AdeRS and AdeN in controlling AdeABC and AdeIJK responses to antibiotics [51. Marchand 2004; 52. Rosenfeld 2012], we examined regulator expression during chloramphenicol stress. Unexpectedly, chloramphenicol did not broadly induce canonical efflux regulators, including *adeRS* and *adeN* (Fig. 5H, Dataset 1). Instead, regulatory changes were selective. The TetR/AcrR-type regulator H0N29_05015, homologous to *A1S_2396* in ATCC17978, was significantly downregulated in Δ*craA* and Δ*adeIJ* strains (Fig. 5E, Dataset 1). Another regulator, H0N29_08595, was repressed in the wildtype and Δ*adeIJ* strains under chloramphenicol (Fig. 5H). In contrast, although several regulators were reduced in Δ*adeAB*, chloramphenicol produced minimal additional transcriptional effects in this mutant (Fig. 5E, 5H)."

5) Line 438. I do not think that the conditions are bacteriostatic. You are using a subinhibitory concentration of chloramphenicol, and the isolates are able to replicate (if they were unable to replicate, you would be at an inhibitory concentration)

We agree that the tested concentration is sub-inhibitory rather than bacteriostatic. The text has been revised to:

Lines 557-559 (marked-up version):

"The robust upregulation of proteostasis-related genes in Δ*adeAB* under sub-inhibitory chloramphenicol exposure is particularly notable…"

6) Line 445. adeAB are on an operon. Was the adeA increase the same as the adeB increase? The same applies for adeIJ.

Thank you for this valuable suggestion. As shown in Dataset 1 and Fig. 5G, both *adeA* and *adeB* were upregulated in the wildtype and Δ*adeIJ* strains in response to chloramphenicol, whereas expression of *adeI* and *adeJ* remained largely unchanged under all conditions, except in comparisons involving the Δ*adeIJ* mutant.

We have included the RNA-seq data (*adeI*, *adeJ*, and *adeK*) in Fig. 5G.

Lines 574-580 (marked-up version):

"Furthermore, induction of *adeA* and *adeB* under chloramphenicol exposure appeared to coincide with increased *craA* expression (Fig. 5G; Supplementary Dataset 1), suggesting a coordinated or potentially complementary efflux activity [28. Foong 2019]. In contrast, adeI and adeJ expression remained largely unchanged across conditions (Fig. 5G, Supplementary Dataset 1)."

7) Is there anything known about the regulator of craA?

We appreciate this insightful question. The regulation of *craA* has not yet been attributed to a specific dedicated transcriptional regulator, but several regulatory features have been reported.

In *A. baumannii* ATCC17978, transcription of *craA* originates from two promoters (TSS-1 and TSS-2), whose usage depends on nutrient availability, indicating environmentally responsive regulation (Kröger 2018). Consistent with this, our previous study showed that *craA* is weakly expressed in LB medium but strongly induced upon chloramphenicol exposure (Foong 2025), suggesting substrate-responsive activation rather than constitutive expression. Together, these findings indicate that *craA* expression is influenced by the physiological state and antibiotic stress rather than a currently defined local regulator, although the specific regulatory factor remains to be identified.

We have clarified this point in the manuscript:

Lines 96-100 (marked-up version):

"Expression of *adeABC* and *adeIJK* ……. whereas the regulatory mechanisms governing *craA* remain less clearly defined, although its expression appears condition-dependent and inducible by both nutrient composition and chloramphenicol exposure [14. Foong 2025, 18. Kröger 2018]."

References:

Kröger et al. 2018. The primary transcriptome, small RNAs and regulation of antimicrobial resistance in *Acinetobacter baumannii* ATCC 17978. *Nucleic Acids Res.* 46, 9684-9698.

Foong et al., 2025. Molecular determinants of substrate specificity in the efflux pump CraA from *Acinetobacter baumannii*. *Microbiol. Spectr.* 13, e0111925.

8) Line 484. The sentence beginning "Despite inactivation of abuO and ompA.." I find it hard to follow. Can you rephrase this please.

We apologize for our lack of clarity. The text has been revised to:

Lines 609-616 (marked-up version):

"Despite previous studies have reported that inactivation of outer membrane proteins (OMP) genes such as *abuO* and *ompA* increases chloramphenicol susceptibility [59. Smani 2014; 60. Srinivasan 2014], our transcriptomic analysis showed that chloramphenicol exposure and efflux pump deletions did not substantially alter the expression of major *OMP genes, including abuO*, *carO*, *ompA, ompW, and omp33–36* (Fig. 5C, 5D, Supplementary Dataset 1)."

9) I would be interested in hearing how the knockout strains accumulate substrate like ethidium bromide compared to wt. Presumably this is older data, but it could still be discussed.

We thank the reviewer for this insightful suggestion. In response, we performed additional ethidium transport experiments to directly evaluate substrate accumulation and efflux capacity in the wildtype and efflux pump knockout strains. These analyses revealed that the Δ*craA* mutant exhibited modestly reduced ethidium efflux compared to the wildtype and RND knockout strains, supporting a functional contribution of CraA to ethidium transport. We also observed a smaller but significant reduction in ethidium efflux in the Δ*adeIJ* mutant relative to wildtype. Although these effects were less pronounced than that observed for chloramphenicol susceptibility, the results are consistent with the role of CraA as a multidrug transporter with overlapping substrate specificity within the *A. baumannii* efflux network. Together, these data provide complementary functional evidence linking CraA activity to intracellular substrate handling and help contextualize the observed phenotypic differences among the efflux mutants.

The ethidium efflux assay has now been incorporated into the revised manuscript.

Method, lines 281-313 (marked-up version):

A detailed description of the ethidium accumulation and efflux assay has been added to the *Materials and Methods* section.

The corresponding results have been included in the Results section as follows:

Lines 408-413 (marked-up version):

"Consistent with previous observations [14. Foong 2025], the Δ*craA* mutant exhibited a modest but statistically significant reduction in ethidium efflux activity compared with the wildtype and RND knockout strains (Supplementary Fig. S1B-S1C), supporting its functional contribution to multidrug efflux. The Δ*adeIJ* mutant likewise displayed a smaller yet significant reduction in ethidium efflux relative to the wildtype (Supplementary Fig. S1C)."

10) Lines 651-654: are there any data on increased expression of T6SS and reduced antimicrobial susceptibility?

We thank the reviewer for this insightful question. Several studies have explored the relationship between type VI secretion system (T6SS) activity and antimicrobial resistance in *A. baumannii*. For example, an analysis of 182 clinical *A. baumannii* isolates reported that T6SS-positive strains exhibited significantly higher resistance rates to multiple antimicrobials, including ceftazidime, imipenem, ciprofloxacin, cefepime, levofloxacin, gentamicin, and sulfamethoxazole, compared with T6SS-negative isolates (Lin 2023). Conversely, T6SS-

negative isolates showed significantly lower resistance rates across these antibiotics. However, other studies have reported contrasting observations, where activation of T6SS was linked to reduced resistance to several clinically relevant antibiotics, including β-lactams, aminoglycosides, erythromycin, and tetracycline (Weber 2015). These divergent observations highlight the complex regulatory interplay between T6SS activity, envelope physiology, and antimicrobial susceptibility. While the association between T6SS activity and antimicrobial resistance remains an area of active investigation, these findings suggest that T6SS may influence antimicrobial resistance in a context-dependent manner.

However, it is important to note that most available studies report correlations based on the presence or expression of T6SS-related genes rather than demonstrating a direct mechanistic link between T6SS expression and altered antimicrobial susceptibility. The resistance phenotypes observed in T6SS-positive or T6SS-negative strains may therefore reflect broader genomic or regulatory differences among clinical isolates rather than a direct role of T6SS in antibiotic efflux or drug inactivation.

Lin 2023. Clinical impact of the type VI secretion system on clinical characteristics, virulence and prognosis of *Acinetobacter baumannii* during bloodstream infection. *Microb Pathog* 182, 106252.

Weber 2015. A multidrug resistance plasmid contains the molecular switch for type VI secretion in *Acinetobacter baumannii*. *Proc Natl Acad Sci USA* 112, 9442–7.

To clarify this point, we have revised the text in the manuscript to acknowledge the reported associations while emphasizing that the causal relationship between T6SS expression and antibiotic susceptibility remains to be fully established.

Revised text added to the manuscript:

Lines 771-778 (marked-up version):

"Repression of the type VI secretion system (T6SS) in Δ*adeAB* is consistent with observations that T6SS inactivation can be associated with enhanced antibiotic resistance [73. Weber 2015] (Fig. 7C, Supplementary Table S5), whereas its activation may increase oxidative sensitivity under Mn^{2+} limitation [46. Bhowmik 2025]. Previous studies have reported that T6SS-positive *A. baumannii* clinical isolates exhibit higher resistance rates to several antimicrobials compared with T6SS-negative isolates. However, whether increased T6SS expression directly contributes to reduced antimicrobial susceptibility or instead reflects broader strain-specific regulatory differences remains unclear."

Additionally, we attempted to construct a knockout of the *tssM* gene, a structural component of the T6SS. However, we observed that inactivation of *tssM* resulted in a growth defect in LB medium, both in liquid culture and on agar plates (Figure 1). While some gene knockouts, such as those in LPS biosynthetic gene cluster, cause growth defects in standard bacterial growth media but are rescued by the addition of specific drugs (polymyxin B), we believe that the growth defect observed in the *tssM* mutant could confound the interpretation of antimicrobial susceptibility assays. Therefore, we have not included the growth curve data for the *tssM* mutant in this manuscript, as it falls outside the current scope of the study.

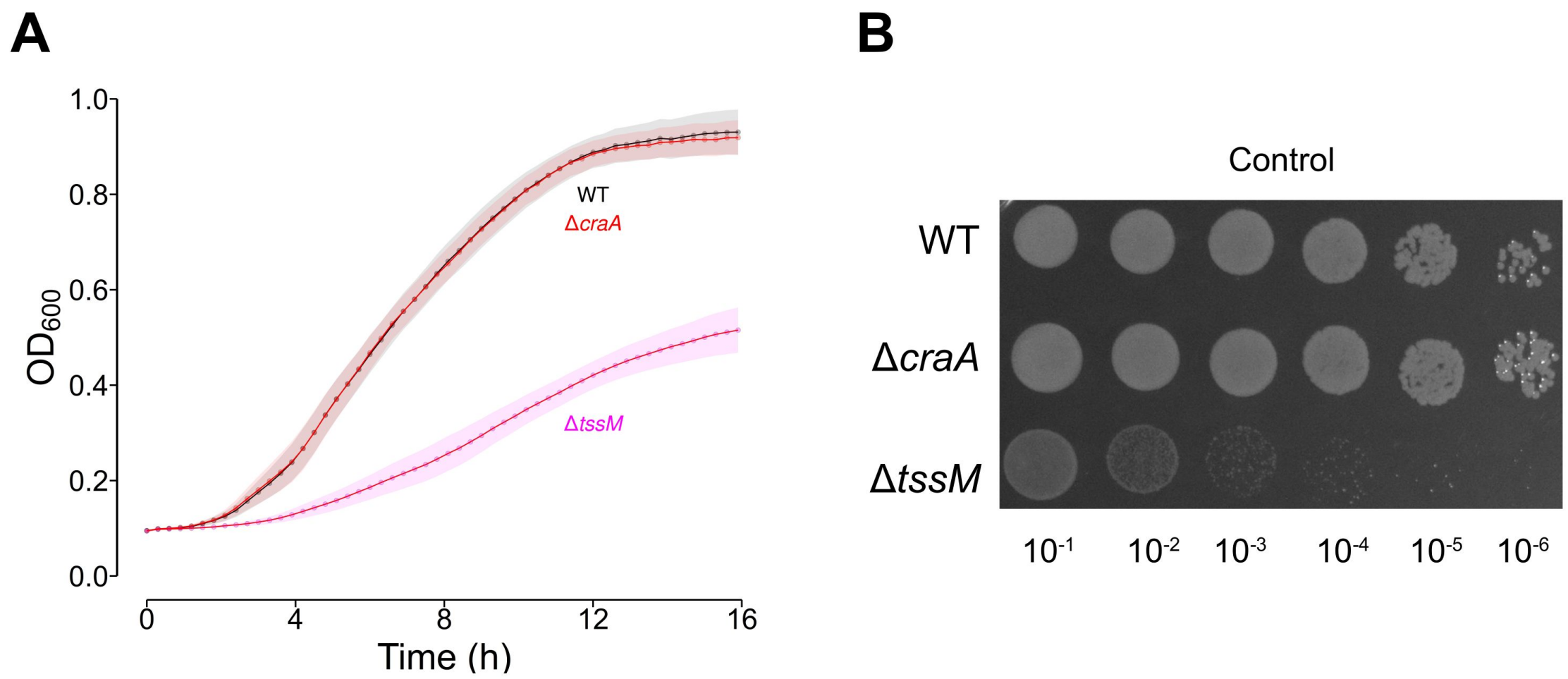

Figure 1. Drug resistance profiles of *A. baumannii* wildtype (WT) and knockout strains. (A) Growth curves in LB. (B) Serially diluted overnight cultures of these strains were spotted on LB agar plates. Experiments were conducted at least three times and the result shown is representative.

11) Table 1. Can you explain the relevance of the bold font

We apologize for our lack of clarity. We have incorporated a statement to explain the meaning of the bold font in Table 1:

Boldface values indicate significant differences in IC_{50} and MIC relative to the wildtype strain (WT).

12) Line 921. "E" is listed twice. I think the second time it should be an "F"

We apologize for this mistake. We have revised the label of the legend.

13) I am struggling to understand figure 2. Can the data be summarised in a less complicated way?

We apologize for the lack of clarity. Figure 2 has been simplified by presenting each strain in separate panels.

14) Figure 7: I really like the concept of the schematic however it is misleading as it suggests that craA acts from the cytosol and crosses over the outer membrane

We agree the original schematic was misleading. The figure has been revised to show CraA transport from the cytoplasm to the periplasm, followed by export through RND pumps. The legend now states:

“The curved arrows on CraA indicate transport of chloramphenicol from the cytoplasm to the periplasm, where RND efflux pumps (AdeABC and/or AdeIJK) subsequently export the drug across the outer membrane.”

Re: mSystems00009-26R1 (**Chloramphenicol Stress Triggers Oxidative Adaptation in *Acinetobacter baumannii* Devoid of RND efflux pumps AdeAB or AdeIJ**)

Dear Dr. Heng-Keat Tam:

Thank you for the privilege of reviewing your work. Below you will find my comments, instructions from the mSystems editorial office, and the reviewer comments.

Your resubmitted manuscript has been really improved. However, I think it is necessary further improve some methodological details and the strongness of discussions based on the obtained results and according to the reviewers' comments

Please return the manuscript within 10 days; if you cannot complete the modification within this time period, please contact me. If you do not wish to modify the manuscript and prefer to submit it to another journal, notify me immediately so that the manuscript may be formally withdrawn from consideration by mSystems.

Revision Guidelines
To submit your modified manuscript, log into the submission site at https://msystems.msubmit.net/cgi-bin/main.plex. Go to Author Tasks and click the appropriate manuscript title to begin. The information you entered when you first submitted the paper will be displayed; update this as necessary. Note the following requirements:

• Upload point-by-point responses to the issues raised by the reviewers in a file named "Response to Reviewers," NOT in your cover letter.
• Upload a compare copy of the manuscript (without figures) as a "Marked-Up Manuscript" file.
• Upload a clean .DOC/.DOCX version of the revised manuscript and remove the previous version.
• Each figure must be uploaded as a separate, editable, high-resolution file (TIFF or EPS preferred), and any multipanel figures must be assembled into one file.
• Any supplemental material intended for posting by ASM should be uploaded with their legends separate from the main manuscript. You can combine all supplemental material into one file (preferred) or split it into a maximum of 10 files with all associated legends included.

For complete guidelines on revision requirements, see our Submission and Review Process webpage. Submission of a paper that does not conform to guidelines may delay acceptance of your manuscript.

Data availability: ASM policy requires that data be available to the public upon online posting of the article, so please verify all links to sequence records, if present, and make sure that each number retrieves the full record of the data. If a new accession number is not linked or a link is broken, provide mSystems production staff with the correct URL for the record. If the accession numbers for new data are not publicly accessible before the expected online posting of the article, publication may be delayed; please contact production staff (mSystems@asmusa.org) immediately with the expected release date.

Publication Fees: For information on publication fees and which article types are subject to charges, visit our website. If your manuscript is accepted for publication and any fees apply, you will be contacted separately about payment during the production process; please follow the instructions in that e-mail. Arrangements for payment must be made before your article is published.

ASM Membership: Corresponding authors may join or renew ASM membership to obtain discounts on publication fees. Need to upgrade your membership level? Please contact Customer Service at Service@asmusa.org.

The ASM Journals program strives for constant improvement in our submission and publication process. Please tell us how we can improve your experience by taking this quick Author Survey.

Thank you for submitting your paper to mSystems.

Sincerely,
Maria Scrascia
Editor
mSystems

Reviewer #1 (Comments for the Author):

While the manuscript has improved substantially, I have these three concerns.
1. Validation of Mutant-Specific Effects
The additional methodological details on mutant construction and genome validation are appreciated. However, the study still

lacks functional complementation or equivalent validation to demonstrate that the observed phenotypes are directly attributable to loss of the targeted efflux pumps rather than secondary regulatory consequences. Reliance on knockout mutants alone limits confidence in causal interpretation, particularly for broad transcriptomic and stress-response phenotypes.

2. Interpretation of Mechanistic Links
Although some statements have been moderated, several conclusions remain stronger than the presented evidence fully supports. In particular, proposed links between efflux pump inactivation and oxidative stress adaptation, metal homeostasis, or coordinated efflux regulation are based largely on transcriptomic associations and indirect phenotypic observations. These interpretations should be framed more cautiously as hypotheses or putative mechanisms unless supported by direct functional validation.

3. Scope and Generalisability of Conclusions
The revised discussion better contextualises compensatory resistance mechanisms, but the broader implications of findings derived from single-gene deletions in one laboratory strain remain somewhat overstated. Additional emphasis on strain specificity, functional redundancy among efflux systems, and limitations of single-mutant transcriptomic analyses would further improve balance and interpretation.

Reviewer #2 (Comments for the Author):

The authors have addressed all my points and I am satisfied with their answers.

We have addressed the comments from the reviewers and the answers given are in blue letter-color (please refer also to the tracked changes documents):

Reviewer #1 (Comments for the Author):

While the manuscript has improved substantially, I have these three concerns.

1. Validation of Mutant-Specific Effects
The additional methodological details on mutant construction and genome validation are appreciated. However, the study still lacks functional complementation or equivalent validation to demonstrate that the observed phenotypes are directly attributable to loss of the targeted efflux pumps rather than secondary regulatory consequences. Reliance on knockout mutants alone limits confidence in causal interpretation, particularly for broad transcriptomic and stress-response phenotypes.

We thank the reviewer for this important point regarding functional validation. We agree that complementation can provide strong evidence for directly linking observed phenotypes to loss of the targeted gene, and we have carefully considered this approach in the context of *A. baumannii* genetic systems.

We acknowledge that chromosomal or integrative complementation approaches are available in *A. baumannii*. However, in our laboratory, complementation systems commonly rely on plasmid-based antibiotic selection markers, most frequently aminoglycosides, which are widely used in *A. baumannii* genetic manipulation (Sykes 2020). It has also been reported that several bactericidal antibiotics used for selection, including aminoglycosides, can induce reactive oxygen species (ROS) as part of their downstream physiological effects (Kohanski 2007). Given that a central focus of this study is the interplay between efflux pump perturbation, chloramphenicol stress, and oxidative responses, the use of antibiotic-based selection during complementation may introduce confounding physiological effects that complicate interpretation of stress-related phenotypes.

For this reason, and to minimize potential confounding factors, we did not include complementation in the present study. Instead, we strengthened the robustness of our conclusion through multiple complementary approaches:

(i) comprehensive whole-genome sequencing confirmation of all mutants, with no off-target mutations detected outside the intended deletions;
(ii) reproducible phenotypic characterization across independent assays, including antimicrobial susceptibility testing, ROS measurements, stress-response assays, ethidium efflux analysis, and transcriptomic profiling;
(iii) careful revision of the manuscript to moderate causal language and ensure appropriate interpretation of knockout-derived phenotypes.

Furthermore, we performed additional RNA-seq analyses to assess transcriptional changes in genes flanking the deletion loci. Specifically, we examined upstream and downstream genes of *adeAB* (*adeR*, *adeS*, H0N29_08685, and H0N29_08690), *adeIJ* (H0N29_03535, *adeK*, H0N29_03555, and H0N29_03560), and *craA* (H0N29_01685, H0N29_01690, *yccS*, and H0N29_01705) relative to the wildtype (refer to Foong-et-al_Supplementary_Dataset1). These analyses indicated no significant transcriptional perturbations in the local genomic

regions, suggesting that the deletions did not substantially affect expression of adjacent genes. Collectively, these observations support that the observed phenotypes primarily attributable to loss of the targeted genes rather than polar effects on neighbouring loci.

Accordingly, we have revised the Conclusion to explicitly highlight the limitations of single-gene knockout approaches, particularly in relation to potential compensatory regulatory effects. We now emphasize that the regulatory response to chloramphenicol in ATCC19606 appears to be strain-specific, likely reflecting functional redundancy among efflux systems, and that extrapolation from single-mutant transcriptomic datasets should be made caution. Overall, the manuscript has been reframed to focus on associations observed in ATCC19606 and to appropriately contextualize the complexity of efflux-mediated stress adaptation. We also clarify that the observed links between efflux regulation, central metabolism, and stress physiology highlight the complexity of intrinsic antibiotic resistance, and underscore the importance of considering both strain-specific variation and efflux system redundancy in *A. baumannii*.

For example,

Lines 735-741 (marked-up version):

"However, the regulatory response to chloramphenicol appeared to be strain-specific in ATCC19606, suggesting functional redundancy among efflux systems and highlighting the limitations of extrapolating findings from single-gene deletion transcriptomic analyses. The observed connections among efflux regulation, central metabolism, and stress physiology point to the complexity of intrinsic antibiotic resistance and highlight the importance of accounting for strain-specific variation and functional redundancy among efflux systems in *A. baumannii*."

References:

Kohanski, 2007. A common mechanism of cellular death induced by bactericidal antibiotics. *Cell* 130, 797-810.

Sykes, 2020. Recent advances in genetic tools for *Acinetobacter baumannii*. *Front. Genet.* 11, 601380.

2. Interpretation of Mechanistic Links
Although some statements have been moderated, several conclusions remain stronger than the presented evidence fully supports. In particular, proposed links between efflux pump inactivation and oxidative stress adaptation, metal homeostasis, or coordinated efflux regulation are based largely on transcriptomic associations and indirect phenotypic observations. These interpretations should be framed more cautiously as hypotheses or putative mechanisms unless supported by direct functional validation.

Thank you for this comment. We have further toned down the discussion and conclusion in the revised manuscript.

For example,

Lines 36-40 (marked-up version):

“Phenotypically, the Δ*adeIJ* strain showed elevated basal and H_2O_2-induced ROS levels, and heightened sensitivity to nitrosative stress and Cu^{2+}, which may suggest a role for AdeIJ in oxidative and metal stress responses.”

Lines 42-46 (marked-up version):

“Collectively, …… while RND transporters such as AdeABC and AdeIJK may also be associated with broader stress adaptation processes, including redox and metal homeostasis, in *A. baumannii*.”

Lines 54-62 (marked-up version):

“In particular, the RND efflux pump AdeIJK may be linked to alterations in cellular responses to oxidative, nitrosative, and metal stress, although the mechanistic basis of this interplay remain to be further investigated. Overall, these findings suggest that efflux pumps may contribute to bacterial resilience beyond drug resistance, providing additional insight into efflux system hierarchy and functional redundancy in *A. baumannii*, and may help inform future studies aimed at understanding persistence and efflux-mediated multidrug resistance mechanisms.”

Lines 131-134 (marked-up version):

“Together, these findings suggest that efflux system perturbation may coincide with changes in metabolic and stress-response networks during adaptation to antibiotic stress in *A. baumannii*.”

Lines 524-527 (marked-up version):

“Furthermore, induction of *adeA* and *adeB* ……, suggesting possible coordinated or complementary efflux activity among these transporters [28. Foong 2019].”

Lines 637-639 (marked-up version):

“Collectively, these findings suggest that disruption of RND and MFS efflux pumps may be accompanied by compensatory physiological or regulatory adaptations that could influence oxidative stress tolerance in *A. baumannii*.”

Lines 651-655 (marked-up version):

Together, …... suggests that loss of AdeIJ may be associated with alterations in copper homeostasis and altered cellular physiology linked to disrupted metal ion regulation.

3. Scope and Generalisability of Conclusions
The revised discussion better contextualises compensatory resistance mechanisms, but the broader implications of findings derived from single-gene deletions in one laboratory strain remain somewhat overstated. Additional emphasis on strain specificity, functional redundancy

among efflux systems, and limitations of single-mutant transcriptomic analyses would further improve balance and interpretation.

We thank the reviewer for this comment. We agree that the broader interpretation of findings from single-gene deletions in a single laboratory strain should be presented with appropriate caution.

In response, we have revised the Discussion and Conclusion to further emphasize,

(i) strain-specific differences in efflux regulation and stress responses,
(ii) the functional redundancy among efflux systems in *A. baumannii*,
(iii) the inherent limitations of inferring system-wide physiological roles from single-mutant transcriptomic analyses.

For example,
Lines 662-665 (marked-up version):

“However, both the response to chloramphenicol and the regulatory response to RND knockout under non-stress conditions were strain-specific, reflecting the consequences of functional redundancy among efflux systems.”

Lines 710-714 (marked-up version):

“Collectively, the …. whereas AdeIJK may contribute to redox and metal homeostasis (Fig. 7), with potential compensatory or strain-specific and condition-specific interactions between RND systems and CraA under antibiotic stress.”

Lines 735-741 (marked-up version):

“However, the regulatory response to chloramphenicol appeared to be strain-specific in ATCC19606, suggesting functional redundancy among efflux systems and highlighting the limitations of extrapolating findings from single-gene deletion transcriptomic analyses. The observed connections among efflux regulation, central metabolism, and stress physiology point to the complexity of intrinsic antibiotic resistance and highlight the importance of accounting for strain-specific variation and functional redundancy among efflux systems in *A. baumannii*.”

Lines 741-743 (marked-up version):

“Future work incorporating combinatorial deletions, regulatory mutants, and clinically derived isolates would help clarify the hierarchy and interplay of efflux systems under antibiotic stress.”

In addition, we have clarified in the title that the interpretation of our findings is specific to *A. baumannii* ATCC19606, to better reflect the scope of the study.

“Chloramphenicol Stress Triggers Oxidative Adaptation in *Acinetobacter baumannii* ATCC19606 Devoid of RND Efflux Pumps AdeAB or AdeIJ”

Reviewer #2 (Comments for the Author):
The authors have addressed all my points and I am satisfied with their answers.

Thank you for this comment.

Re: mSystems00009-26R2 (**Chloramphenicol Stress Triggers Oxidative Adaptation in *Acinetobacter baumannii* ATCC19606 Devoid of RND efflux pumps AdeAB or AdeIJ**)

Dear Dr. Heng-Keat Tam:

Your manuscript has been accepted and I am forwarding it to the ASM production staff for publication. Your paper will first be checked to make sure all elements meet the technical requirements. ASM staff will contact you if anything needs to be revised before copyediting and production can begin. Otherwise, you will be notified when your proofs are ready to be viewed.

Data Availability: ASM policy requires that data be available to the public upon online posting of the article, so please verify all links to sequence records, if present, and make sure that each number retrieves the full record of the data. If a new accession number is not linked or a link is broken, provide production staff with the correct URL for the record. If the accession numbers for new data are not publicly accessible before the expected online posting of the article, publication may be delayed; please contact ASM production staff immediately with the expected release date.

Publication Fees: For information on publication fees and which article types have charges, please visit our website. We have partnered with Copyright Clearance Center (CCC) to collect author charges. If fees apply to your paper, you will receive a message from no-reply@copyright.com with further instructions. For questions related to paying charges through RightsLink, please contact CCC at ASM_Support@copyright.com or toll free at +1-877-622-5543. CCC makes every attempt to respond to all emails within 24 hours.

ASM Membership: Corresponding authors may join or renew ASM membership to obtain discounts on publication fees. Need to upgrade your membership level? Please contact Customer Service at Service@asmusa.org.

Embargo Policy: A press release may be issued as soon as the manuscript is posted on the mSystems Latest Articles webpage. The corresponding author will receive an email with the subject line "ASM Journals Author Services Notification" when the article is available online.

Cover Image Submissions: If you would like to submit a potential Cover Image, please email a file and a short legend to msystems@asmusa.org. Please note that we can only consider images that (i) the authors created or own and (ii) have not been previously published. By submitting, you agree that the image can be used under the same terms as the published article. Image File requirements: TIF/EPS, 7.5 inches wide by 8.25 inches tall (at least 2,250 pixels wide by 2,475 pixels tall), minimum 300 dpi resolution (600 dpi preferred), RGB, and no figure elements, e.g., arrows or panel labels. The legend should be a short description of the image, 1-2 sentences recommended. Please download and use this interactive template in Adobe to ensure that your proposed cover image meets our size requirements (https://journals.asm.org/pb-assets/pdf-text-excel-files/ASM-Interactive-Sizing-Cover-Template-1715689791.pdf).

Thank you for submitting your paper to mSystems.

Sincerely,
Maria Scrascia
Editor
mSystems

Reviewer #1 (Comments for the Author):

The authors have addressed most of the concerns raised in the previous review. The manuscript has been substantially improved through the inclusion of additional methodological details, strengthened validation of the mutant strains, and more cautious interpretation of the findings. However, a few statements in the Discussion still imply mechanistic relationships that are not directly supported by the data (e.g., linking transcriptional changes to oxidative stress tolerance and inferring changes in intracellular glutamate and arginine pools without direct measurements). These statements should be further moderated. In addition, a few minor grammatical and typographical errors should be corrected.
